# Comparative genomic analysis and evolution of the T cell receptor loci in the opossum *Monodelphis domestica*

**DOI:** 10.1186/1471-2164-9-111

**Published:** 2008-02-29

**Authors:** Zuly E Parra, Michelle L Baker, Jennifer Hathaway, April M Lopez, Jonathan Trujillo, Alana Sharp, Robert D Miller

**Affiliations:** 1 Center for Evolutionary and Theoretical Immunology and Department of Biology, University of New Mexico, Albuquerque, NM 87131, USA

## Abstract

**Background:**

All jawed-vertebrates have four T cell receptor (TCR) chains: alpha (TRA), beta (TRB), gamma (TRG) and delta (TRD). Marsupials appear unique by having an additional TCR: mu (TRM). The evolutionary origin of TRM and its relationship to other TCR remain obscure, and is confounded by previous results that support TRM being a hybrid between a TCR and immunoglobulin locus. The availability of the first marsupial genome sequence allows investigation of these evolutionary relationships.

**Results:**

The organization of the conventional TCR loci, encoding the TRA, TRB, TRG and TRD chains, in the opossum *Monodelphis domestica *are highly conserved with and of similar complexity to that of eutherians (placental mammals). There is a high degree of conserved synteny in the genomic regions encoding the conventional TCR across mammals and birds. In contrast the chromosomal region containing TRM is not well conserved across mammals. None of the conventional TCR loci contain variable region gene segments with homology to those found in TRM; rather TRM variable genes are most similar to that of immunoglobulin heavy chain genes.

**Conclusion:**

Complete genomic analyses of the opossum TCR loci continue to support an origin of TRM as a hybrid between a TCR and immunoglobulin locus. None of the conventional TCR loci contain evidence that such a recombination event occurred, rather they demonstrate a high degree of stability across distantly related mammals. TRM, therefore, appears to be derived from receptor genes no longer extant in placental mammals. These analyses provide the first genomic scale structural detail of marsupial TCR genes, a lineage of mammals used as models of early development and human disease.

## Background

The hallmarks of the vertebrate adaptive immune system are antigen specific receptors, the T cell receptors (TCR) and immunoglobulins (Ig) encoded by genes that undergo somatic DNA recombination to generate diverse binding specificities. The TCR are expressed by thymus-derived lymphocytes (T cells) that play a major role in regulation and effector functions of immune responses. Each T cell expresses a unique TCR that binds a specific antigen resulting in the activation of an immune response [1,2]. TCR are heterodimers comprised of either alpha (TRA) and beta (TRB) or gamma (TRG) and delta (TRD) combinations, respectively. These two combinations define the two major lineages of T cells: αβT cells and γδT cells [3,2]. αβT cells typically recognize peptide antigens presented on major histocompatibility complex (MHC) encoded molecules. In contrast γδT cells have been shown to be either MHC restricted or in some cases, similar to Ig, able to bind free antigen [4]. Ig are expressed by antibody forming cells (B cells), which produce both a membrane bound form of Ig that comprises the B cell receptor (BCR) and a soluble form that is free antibody. Like TCR, Ig are made up of two different chain types, a heavy (IgH) and light (IgL) chain. TCR and Ig chains both contain variable domains that bind the antigen and membrane-proximal constant (C) domains. It is the variable domains that are encoded by gene segments that undergo somatic recombination to generate diversity in binding specificity. The gene segments encoding the variable domains of TRA, TRG and IgL chains are the variable (V) and joining (J) gene segments, while the variable domains of TRB, TRD and IgH chains are encoded by exons assembled from V, diversity (D) and J gene segments [5]. Recombination of these gene segments takes place in the thymus for developing T cells and adult bone-marrow for developing B cells [5].

Of the immunoglobulin superfamily (IgSF) members, the TCR and Ig are each other's nearest relatives, however there are dissimilarities to their genetic structure and evolutionary history [6,7]. For example all jawed-vertebrates appear to contain the same four homologous TCR isotypes: TRA, TRB, TRG, and TRD [8]. In contrast there is variability in the number and class of Ig isotypes in different vertebrate lineages [9,10]. In addition the organization of TCR loci appears to be more conserved than Ig. For example, in cartilaginous fish most Ig loci are organized as multiple, unlinked clusters of [V-(D)-J-C], limiting the combinatorial usage of their gene segments [11]. Whereas, in bony fish and tetrapods the majority of Ig loci are organized in the translocon style of Vn-(D)n-Jn-Cn [11]. TCR loci tend to be organized in the translocon style in all lineages. These differences between TCR and Ig genes are likely the result of dissimilar selection forces on the two different antigen receptor systems and have made determining the evolutionary relationship of the TCR and Ig chains to each other unclear [12].

The relationship between Ig and TCR is further muddled by the recent discoveries in marsupials and sharks of TCR loci that appear to be hybrids between ancestral Ig and TCR loci [13,14]. In marsupials, a mammalian lineage that diverged from eutherians (placental mammals) 186 to 193 million years ago (MYA), a new fifth TCR chain, named TCR mu (TRM) has been identified [14,15]. Unlike the conventional TCR, TRM has a tandem cluster organization and its origins appear to have involved a recombination between and ancestral TCR locus, most likely a *TRD *and *IgH*. *TRM *appears analogous to an unusual shark TCR called *NAR-TCR*, which utilizes the C regions of TRD and upstream Ig-like V regions [13]. Both TRM and NAR-TCR are expressed in an atypical TCR isoform that contains double variable domains. Marsupial TRM and shark NAR-TCR, however, are not orthologous but rather the product of convergent evolution generating common features [14]. Nonetheless the presence of TCR with these features in both marsupials and cartilaginous fish, make it likely analogous TCR will be found in other vertebrate lineages and illustrate a level of plasticity in TCR evolution heretofore unrealized.

The availability of the first completely sequenced marsupial genome provides the opportunity to investigate the evolutionary origins of TRM and its relationship to the conventional TCR in mammals [16]. Towards this aim we have determined the complete genomic organization, content and evolution of the loci encoding both the conventional TCRs (*TRA, TRB*, *TRG *and *TRD*) and the recently discovered *TRM *locus in the opossum *Monodelphis domestica*. In addition, these analyses provide a level of detail for the TCR genes of a marsupial that has only been available for a limited number of eutherians such as human and mouse.

## Results and Discussion

### *TRA/D *locus

**Figure 1:**
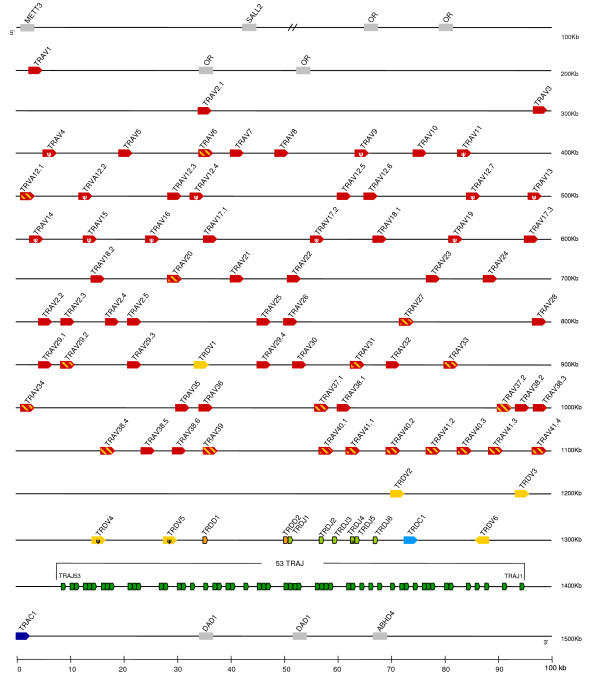
Map of the opossum *TRA/TRD *locus. *TRAV *(red), *TRDV *(yellow), *TRDJ *(light green), *TRAJ *(dark green) and *TRDD *(orange) gene segments are shown numbered by their location in order across the locus. TRV segments are designated with the subgroup number followed by a period and a designated number according to their location. *TRA *segments that were also found expressed with TRDC, and therefore TRA/DV, have diagonal yellow stripes. *TRDC *(light blue) and *TRAC *(dark blue) are also indicated. Transcriptional orientation is indicated by the direction of the arrow on each segment. Presumptive pseudogenes are indicated with a ψ. Syntenic genes discussed in the text are indicated in gray.

**Figure 2:**
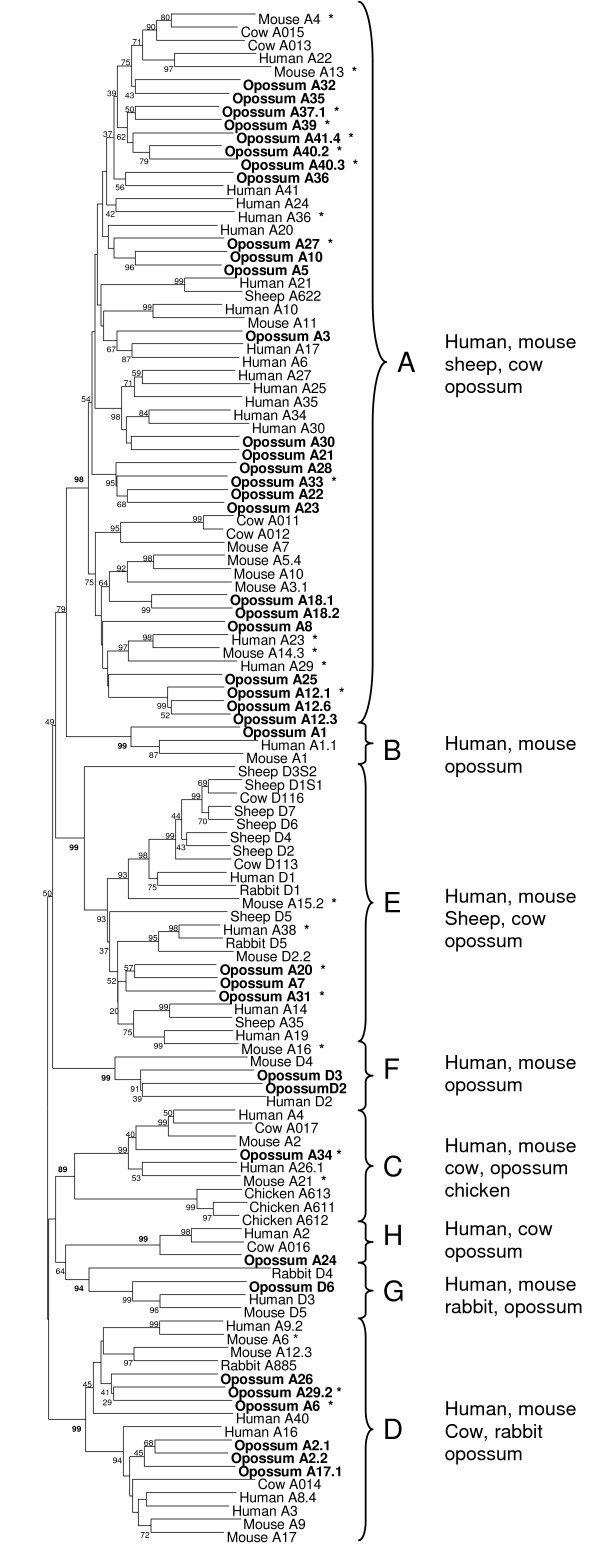
Phylogenetic tree of the *TRAV*, *TRDV *and *TRA/DV *gene segments from mammalian and avian species using the neighbor joining method. Opossum *TRV *sequences are indicated in bold. Mammalian gene segments that are known to be expressed as *TRA/DV *are indicated with an asterisk. *TRV *genes fall into eight groups (A-H, indicated with braces) which indicate the evolutionary relationship of these genes. Branch supports are indicated as the percentage of trees based on 1000 bootstrap replicates.

Previous physical mapping of the TCR loci in the opossum revealed that the *TRA *and *TRD *were co-localized on chromosome 1p [17]. Analysis of the opossum whole genome sequence confirmed that the *TRD *genes are clustered within the *TRA *locus resembling the organization of *TRA/D *observed in eutherians and birds (Figure 1) [18-20]. In opossum, the *TRA/D *locus spans approximately 1.3 Mb, making it intermediate in size compared to that of human at 1 Mb and mouse at 1.65 Mb [21,22]. To investigate the degree of genomic conservation in this region we identified the genes syntenic to opossum *TRA/D *locus and compared these to the available genomic data for mouse, human, cow and chicken using the current ENSEMBL databases for each species [23]. At the 5' end of the opossum *TRA/D *locus are the methyl-transferase like 3 (*METTL3*), zinc finger protein (*SALL2*) and several olfactory receptor (*OR*) loci that have conserved synteny in human, mouse, and cow, but not chicken (Figure 1 and Additional file 1). However these genes are not immediately flanking the cow *TRA/D *locus. Also conserved at the 5' end of the *TRA/D *locus in opossum, human, and mouse are TRA V genes (*TRAV*) interspersed with the *OR *loci. In opossum and mouse there is only one *TRAV *segment interspersed with the *OR*, whereas in human there are two at this location (Figure 1) [22]. These *TRAV *gene segments (opossum *TRAV1*, mouse *TRAV1*, and human *TRAV1.1 *and *1.2*) appear orthologous in phylogenetic analyses forming their own distinct group (group B) in a tree of *TRAV *and *TRDV *sequences (Figure 2). The 3' end of the *TRA/D *locus appears to be the most conserved across species since many of the loci have conserved synteny in both mammals and birds. The opossum has two copies of the *defender against cell death gene 1 *(*DAD1*) also found at the 3' end of the human, mouse, cow and chicken *TRA/D *loci. In those eutherian mammals examined the position of the *abhydrolase domain-containing protein 4 gene *(*ABHD4*) is also conserved (Figure 1 and Additional file 1). The opossum *TRDV6 *gene segment further illustrates the conservation of the *TRA/D *locus across mammals. This gene segment is in an inverted orientation and located downstream of the TRD C (*TRDC*). A clear ortholog of *TRDV6 *is found in both human and mouse with the same location and reading orientation and these gene segments from all three species fall into the same phylogenetic clade (see below, Figure 2). These results all support the overall organization of the mammalian *TRA/D *loci and their flanking genomic regions being highly conserved over a span of at least 186 My and as long as 300 My in some cases [15,24].

Overall the organization and complexity of gene segments within the opossum *TRA/D *locus is similar to that of human and mouse. There are 74 total V segments (*TRAV *plus *TRDV*) in opossum, a number intermediate to that of human and mouse (Table 1). In human and mouse the V gene segments are either *TRAV *or *TRDV*, in other words used in TRA or TRD chains respectively, or in some cases specific V segments have been found expressed in either TRA or TRD chains and have been designated *TRA/DV*. The category a particular V segment falls into is historically defined by a number of criteria. One criterion is nucleotide similarity to V segments defined already in other species; by this criterion there are 68 opossum *TRAV *and six *TRDV *segments when compared with human and mouse. These two groups, with a single exception, are spatially separated in the *TRA/D *locus with the *TRAV *segments at the 5' end and the *TRDV *at the 3' end of the locus. The exception is a single *TRDV *segment (*TRDV1*) interspersed with the TRAV segments (Figure 1). Of the 68 *TRAV *and 6 *TRDV*, 12 and 2 respectively appear to be pseudogenes due to absence of a complete open reading frame (ORF) (Figure 1, Table 1). This is a ratio of functional to non-functional gene segments comparable to human and mouse (Table 1).

**Table 1:** Number of TRV, TRD, TRJ and TRC genes found in human, mouse and opossum. The numbers of functional genes are indicated in parenthesis.

**Species\TCR**	**Human**^†^	**Mouse**^†^	**Opossum**
TRAV*	54 (44–47)	98 (73–84)	68 (56)
**TRAJ**	61 (50)	60 (38)	53 (53)
**TRAC**	1 (1)	1 (1)	1 (1)
**TRDV**	3 (3)	6 (5)	6 (4)
**TRA/DV**	5	10	20
**TRDD**	3	2	2
**TRDJ**	4 (4)	2 (2)	6 (5)
**TRDC**	1 (1)	1 (1)	1 (1)
**TRBV**	64–67 (40–48)	35 (21–22)	36 (27)
**TRBD**	2	2	4 (3?)
**TRBJ**	14 (12–13)	14 (11)	18 (18)
**TRBC**	2 (2)	2 (2)	4 (4)
**TRGV**	12–15 (4–6)	7 (7)	9 (9)
**TRGJ**	5 (5)	4 (4)	7 (5)
**TRGC**	2 (2)	4 (3)	1 (1)

* Include the TRAV that are TRA/D.

^† ^[21]

To determine which of the 74 V gene segments are also used as *TRA/DV *we performed RT-PCR on 23 day old thymus RNA using combinations of primer pairs specific for each of the V segment subgroups (see below) paired with either TRA or TRD C regions (*TRAC *and *TRDC *respectively) (not shown). This age was chosen as an early age where the thymus is fully mature [25]. Nineteen of the *TRAV *segments and all four of the functional *TRDV *segments were found expressed with both TRAC and TRDC resulting in a total of 23 apparent *TRA/DV *segments (Figure 1 and Table 1). The number of opossum *TRA/DV *may be an underestimate since it is possible some *TRAV *may be rarely expressed with TRDC or may appear at different times during development than was examined. Alternatively this number could be an overestimate as well, since it is possible that some combinations are negatively selected in the thymus and not used in the periphery. Either way this appears to be a comparatively high number of *TRA/DV *segments in the opossum relative to human and mouse (Table 1).

V gene segments evolve by gene duplication and deletion resulting in degrees of relatedness amongst segments [26]. These are defined as subgroups with the segments belonging to the same subgroup by having 80% or greater nucleotide identity. By this criterion the current 68 *TRAV *segments can be placed into 41 subgroups, where the nucleotide identity between subgroups ranges from 32.1 to 79.5%. The six *TRDV *gene segments were sufficiently different, with nucleotide identity ranging from 33.6 to 61.9%, that each belonged to its own distinct subgroup. Phylogenetic analyses using *TRAV *and *TRDV *segments were performed to elucidate their evolutionary relatedness. Opossum sequences were compared with sequences from human, mouse, rabbit, cow, sheep, and chicken using the same dataset as used by Su *et al*. [27] to define the major phylogenetic groups (Figure 2). Eight groups of V gene segments (designated A through H) with bootstrap values greater than 89% emerged from the inclusion of the marsupial sequences (Figure 2). All eight contain opossum sequences (Figure 2). Four of these, groups A through D, were defined previously and only included TRAV or TRAV/D [27]. However, the addition of opossum sequences revealed four new V groups (E through H) not previously recognized or requiring reevaluation. Group E contains TRAV, TRDV and TRA/DV sequences; groups F and G only TRDV sequences; and group H TRAV and TRA/DV sequences. The addition of the opossum sequences to this analysis substantiate the statement that all these subgroups were present in the common ancestor of amniotes and that some species have lost segments that belong to different subgroups [27].

These analyses also allow us to evaluate the evolution of mammalian V segments that can be utilized with either TRA or TRD chains over a larger time-span than has been available previously. TRA/DV sequences clearly belong to different groups rather than forming a monophyletic cluster (Figure 2) and, as observed for human and mouse, TRA/DV regions are dispersed throughout the opossum *TRA/D *locus, although most are located towards the 3' end of the locus (Figure 1). Considering that αβ and γδT cells recognize potentially very different antigens these results continue to support the high level of plasticity in V gene utilization at this locus.

The number and complexity of D and J gene segments in the opossum *TRA/D *locus is also comparable to that of human and mouse (Figure 1, Table 1). Encoding TRD chains are at least two D and six J gene segments (*TRDD *and *TRDJ *respectively), all of which are located upstream of a single *TRDC*. There are at least 53 TRAJ segments located between the *TRDC *and *TRAC *all of which appear to be functional by the criteria defined above for the V gene segments (Figure 1, Table 1).

**Figure 3:**
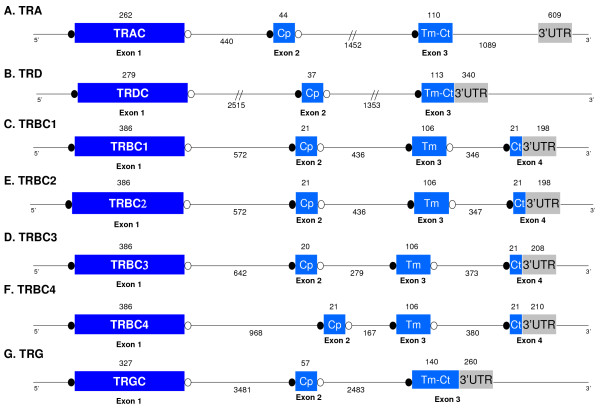
Exon and intron organization of the opossum *TRC *genes. Numbers above and below indicate the length of the exons and introns in base pairs, respectively. Indicated in white circles are the donor splice sites and in black the acceptor splice sites. **A**: *TRAC*; **B**: *TRDC*; **C**: *TRBC1*; **D**: *TRBC2*; **E**: *TRBC3*; **F**: *TRBC4*; **G**: *TRGC*.

The opossum TRAC and TRDC regions are encoded by three exons (Figure 3A, 3B) that, as reported previously, encode residues conserved in other species [28,29]. For both TRAC and TRDC, exon 1 encodes an IgSF domain, which contains two cysteine residues that form the intra-chain disulfide bond. Exon 2 encodes the connecting peptide (Cp) containing the cysteine residue involved in the inter-chain disulfide bond. Exon 3 encodes the transmembrane (Tm) and a short cytoplasmic (Ct) region (Figure 3A, 3B). In the TM region of both TRA and TRD there are two hydrophilic residues (lysine and arginine) that are conserved in other species and that are important for interaction with other dimers from the TCR complex [30]. There are five potential N-glycosylation sites in the opossum TRAC and two in the TRDC [28,29].

### *TRB *locus

**Figure 4:**
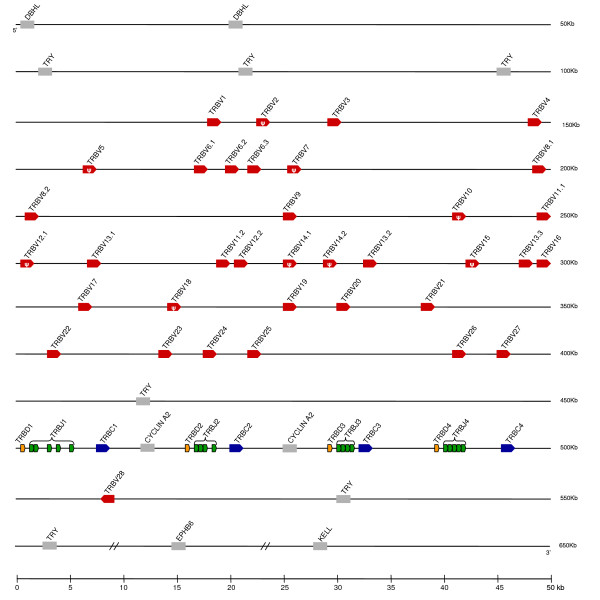
Map of the opossum *TRB *locus. *TRBV *(red), *TRBD *(orange), *TRBJ *(green) and *TRBC *(blue) are indicated. Transcriptional orientation, pseudogenes, and syntenic genes discussed in the text are indicated as in Figure 1.

**Figure 5:**
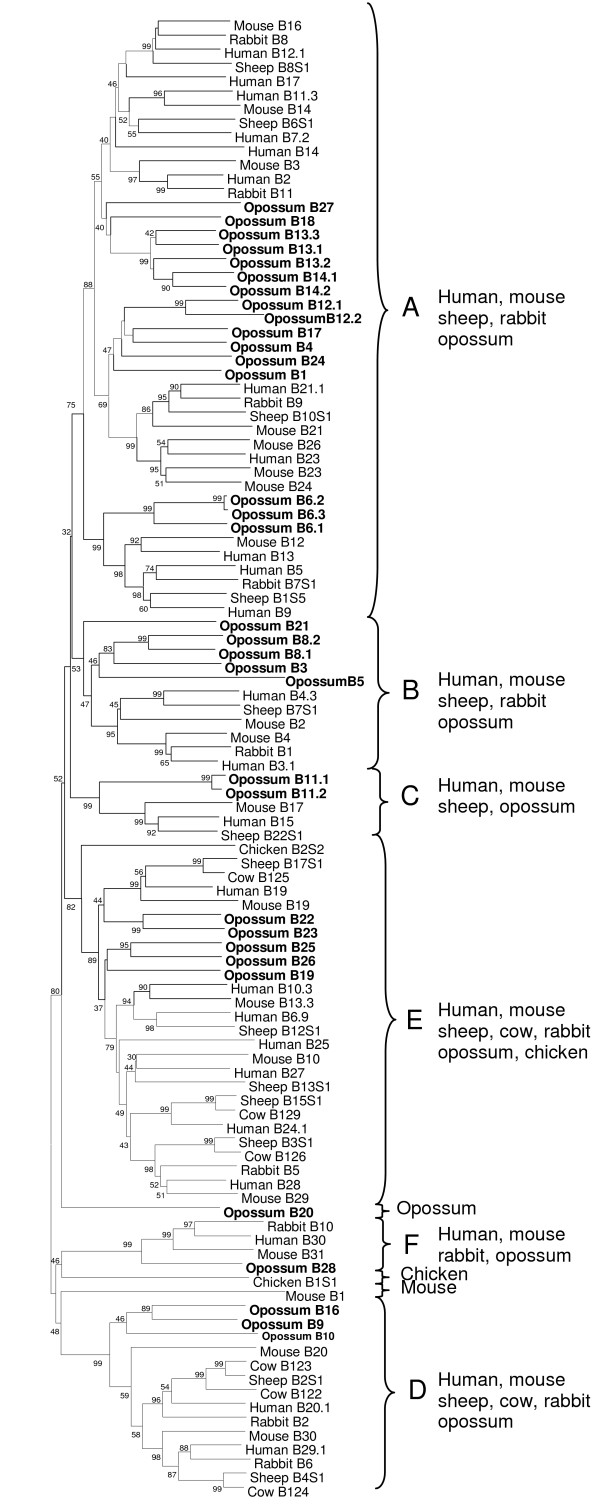
Phylogenetic tree of the *TRBV *gene segments from mammalian and avian species using the neighbor joining method. Opossum *TRBV *sequences are indicated in bold. The percent bootstrap values based on 1000 replications are indicated. Major phylogenetic groups (A-F) are indicated with braces.

*TRB *has been physically mapped to chromosome 8q in the opossum [17]. The opossum *TRB *locus spans 400 kb making it smaller in size than its human and mouse homologues that are about 650 kb each (Figure 4) [31,22]. Genes syntenic the opossum *TRB *locus are also conserved across human, mouse, cow and chicken. These include the trypsinogen genes (TRY) found at the 5' and 3' ends of the *TRB *locus and intermixed between the *TRBV *and *TRBC *gene segments (Figure 4) [22]. The mono-oxygenase DBH-like 2 (*DBHL*) is found at the 5' end of the locus in the opossum, similar to human, mouse, and cow, but not in chicken. Genes such as *kell blood group glycoprotein *(*Kel*) and *ephrin type-b receptor 6 precursor *(*EPHB6*) found at the 3' end of the opossum *TRB *locus have conserved synteny in mammals and chicken (Figure 4). As with *TRA/D*, the *TRB *locus organization is highly conserved between opossum and eutherians. This is further illustrated by a *TRBV *segment (TRBV28 in opossum) located at the 3' end of the locus that is in the reverse orientation relative to the other gene segments. A clear orthologue of this gene segment is present in human (*TRBV30*) and mouse (*TRBV31*) making this an ancient arrangement (Figure 4 and group F in Figure 5).

There are 36 opossum *TRBV *segments (Figure 4, Table 1) and these can be grouped in 28 subgroups based on nucleotide identity with the value between subgroups ranging from 38.5 to 68.5%. Compared with other TCR, the *TRB *locus in eutherians appears to contain a higher number of V pseudogenes, where 19% and 34% of *TRBV *are pseudogenes in human and mouse, respectively. This pattern appears to hold for the opossum since nine of the 36 *TRBV *segments (25%) appear to be pseudogenes (Table 1).

Phylogenetic analyses of the TRBV regions from different mammals and chicken reveal that the opossum TRBV regions are very diverse with six groups (A to F) identified (Figure 5). Human, mouse and opossum TRBV sequences are found in all six groups, supporting their presence before the divergence of marsupial and eutherian mammals (Figure 5) [27]. Three sequences chickenB1S1, mouseB2 and opossumB20 did not cluster with any other sequence, nor with each other, and therefore not included within any of the groups.

As in human and mouse, the opossum TRB D, J, and C genes are organized in tandem cassettes. The human and mouse *TRB *locus contains two of such D-J-C cassettes while the opossum has four (Figures 3C–3F and 4). In the opossum, each cassette contains a single *TRBD*, four or five *TRBJ*, and a single *TRBC*. All four cassettes appear to be functional and *TRBJ *gene segments from each have been found in TRB transcripts (data not shown).

The four opossum TRBC regions are very similar to each other at the nucleotide level and in intron – exon organization. Each TRBC region is encoded by four exons (Figure 3C–F). Exon 1 encodes the immunoglobulin domain which contains two conserved cysteines residues important for the intra chain disulfide bond formation. There are three potential N-glycosylation sites in exon 1. Exon 1 of TRBC1, TRBC2 and TRBC3 have 100% nucleotide identity and TRBC4 differs by only a single non-synonymous nucleotide substitution (K10E) that encodes a lysine (AAA) instead of the glutamate (GAA). Exon 2 encodes the Cp and it contains a conserved cysteine residue used for the inter chain disulfide bond (Figure 3C–F). The Cp from the four opossum TRB share more than 82.6% nucleotide identity. Exon 3 encodes the Tm region and contains a lysine residue involved in the interaction with the CD3 complex. The Tm regions are much conserved among the four opossum TRBC, sharing greater than 85.1% nucleotide identity. Exon 4 encodes the cytoplasmic region and it also includes 3' untranslated region.

Due to the high degree of sequence similarity among the four cassettes described above it is difficult to fully reconstruct the duplication events that led to the current arrangement in the opossum. However, cassettes 2 and 3 share two characteristics indicating they are derived from a relatively recent tandem duplication. First of all, both cassettes are nearly identical in nucleotide sequence over a 12.8 kb region that extends from 4.8 kb upstream of a non-functional copy of *cyclin A2 *including the gene segments *TRBD, TRBJ *to 100 bp downstream of *TRBC *(Additional file 2). Secondly both cassettes 2 and 3 have a *cyclin A2 *gene 5' of the D-J-C gene segments, which is not present in the other two cassettes (Figure 4). *Cyclin A2 *is also associated with the cow TRB locus but is not in human, mouse or chicken. This is consistent with the *cyclin A2 *gene being inserted near the TRB D-J-C cassettes prior to the divergence of marsupials and eutherians, with subsequent loss in some eutherian species such as human and mouse.

### *TRG *locus

**Figure 6:**
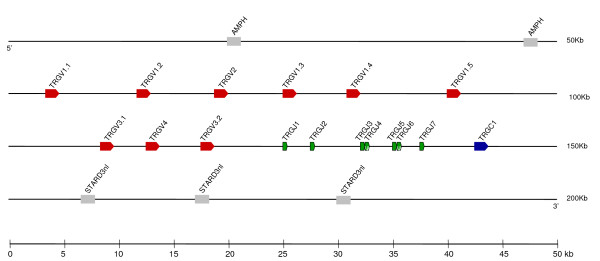
Map of the opossum *TRG *locus. *TRGV *(red), *TRGJ *(green) and *TRGC *(blue) are indicated. All other designations are as in Figure 1.

*TRG *has been physically mapped to chromosome 6q in the opossum [17]. As in human and mouse the *TRG *locus is the smallest and least complex of the three conventional TCR loci. From the most 5' V to the 3' untranslated region (UTR) of the single C region, the opossum *TRG *locus spans only approximately 90 kb (Figure 6), smaller than that in human (150 kb) and mouse (205 kb) [32,33]. The opossum *TRG *locus has a translocon organization, which is different from that present in human and mouse (Figure 6). The human *TRGV *segments are upstream of two *TRGJ-TRGC *cassettes, while in mouse there are four cassettes that contain *TRGV, TRGJ *and *TRGC *gene segments [34,33]. Even though the organization of V, J and C gene segments appears different between human, mouse and opossum, the genes flanking this locus are conserved among these species (Figure 6). *Amphiphysin *(*AMPH*) and the *related to steroidogenic acute regulatory protein D3-N-terminal like *(*STARD3NL*) are found at the 5' and 3' end of the *TRG *locus respectively (Figure 6)[22]. Both *AMPH *and *STARD3NL *are also associated with the *TRG *locus in cow and chicken, although their locations appear changed when searching in their current assembled genomes [35]. In cows and sheep there are two TRG loci, TRG1 and TRG2 [36,37]. To determined if the opossum also has more than one TRG locus we examined its genome thoroughly by performing BLASTN searching of the entire MonDom5.0 assembly using the TRG cDNA and genomic sequences. Only a single TRG locus was identified and this corresponds to the locus we mapped previously to chromosome 6q (Figure 6) [17].

**Figure 7:**
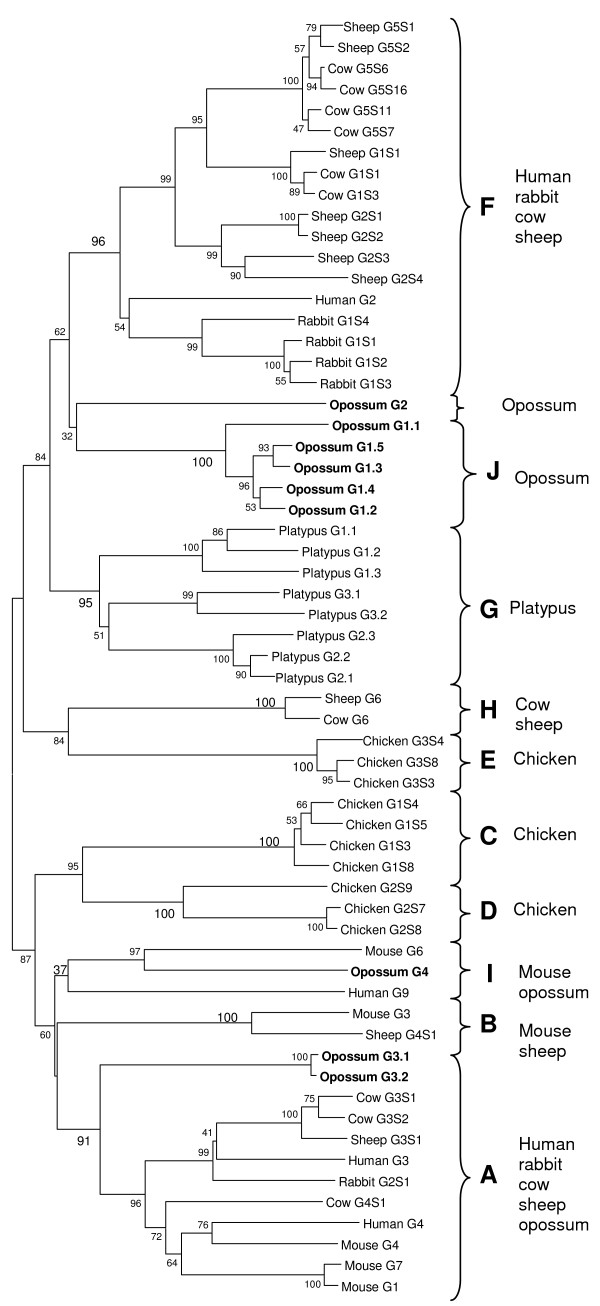
Phylogenetic tree of the *TRGV *gene segments from mammalian and avian species using the neighbor joining method. Opossum *TRGV *sequences are indicated in bold. The percent bootstrap values based on 1000 replications are indicated. Major phylogenetic groups (A-J) are indicated with braces.

There are nine *TRGV *gene segments present in the opossum and these are divided into four subgroups based on nucleotide identity (Figure 7). All *TRGV *segments appear to be functional, and have been found expressed in the thymus (not shown). The number of *TRGV *segments is similar to that in mouse where there are seven V segments, all of which are functional. In human there are fourteen V segments, but only six are functional (Table 1).

Previously, phylogenetic analyses of the mammalian and avian *TRGV *segments revealed the presence of eight ancient groups [38]. Addition of the opossum TRGV sequences to these analyses revealed two additional groups, group I and J. Group I contains human, mouse and opossum TRGV4. Support for this group is low (Figure 7), however it is likely that these three sequences from opossum, human and mouse are derived from the same ancestral gene since they also have similar RSS sequences (data not shown). The new group J contains only the members of the opossum TRGV1 subgroup (Figure 7), which is related to the previously defined group F that contains sequences from human, rabbit, sheep and cow [38]. This relationship is not well supported however, and two groups may not have arisen from a common ancestral gene segment (Figure 7). Opossum TRGV3 segments group in a previously defined group A. The single member of opossum TRGV2 subgroup does not clearly cluster with any existing group (Figure 7).

There is only a single opossum TRGC region (Figure 3G and 6) in contrast to four in mouse (three of which are functional) and two in human (both functional) (Table 1). The opossum TRGC is encoded by three exons. Exon 1 encodes the immunoglobulin domain and contains a single N-glycosylation site. An unusual characteristic described previously in marsupials was the absence of the second cysteine residue required for the formation of intrachain disulfide bond in the TRGC region [38]. Exon 2 encodes the Cp and exon 3 encodes the Tm, Ct and 3'UTR regions (Figure 3G).

### *TRM *locus

**Figure 8:**
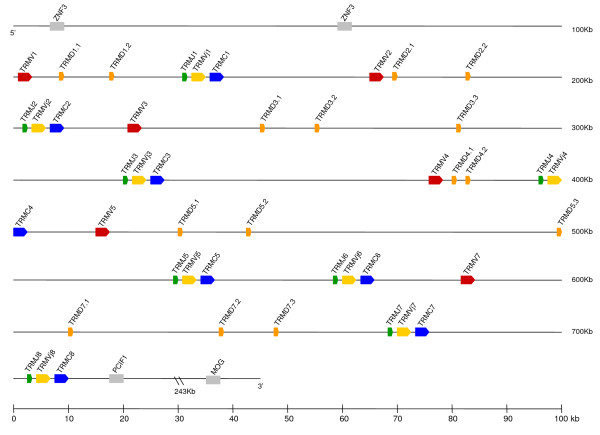
Map of the opossum *TRM *locus. *TRMV *regions are colored in red, *TRMD *in orange, *TRMJ *in green, *TRMVj *in yellow and *TRMC *in blue. Clusters are numbered starting at the 5' end with V, D, J gene segments and C regions numbered according to the cluster to which they belong. All other designations are as in Figure 1.

The *TRM *locus has been described so far only in marsupials and is located on chromosome 3q in the opossum [14]. Homologs to *TRM *have yet to be found in any eutherian mammal examined so far (results not shown). Previous analyses of the *TRM *locus were consistent with *TRM *being a hybrid locus generated by recombination between ancestral Ig and TCR genes [14]. To examine this hypothesis further we analyzed the genes flanking the *TRM *locus to look for any evidence of this recombination. On the immediate centromeric, 5' side of *TRM *are three zinc finger protein genes of the C2H2 type (*ZNF3*) (Figure 8 and Additional file 1). Unfortunately these share similarity to human and mouse *ZNF3 *genes on several chromosomes making orthology difficult to establish (data not shown). On the telomeric, 3' side of TRM are genes encoding *Speckle type POZ-like protein *(*PCIF1*-like) and *Myelin Oligodendrocyte Glycoprotein *(*MOG*). In both cases *PCIF1 *and *MOG *have paralogous copies in the opossum genome and the paralogue syntenic to *TRM *is the least similar to the eutherian homologue. None of the genes flanking the opossum TRM locus have conserved synteny in human and mouse making it difficult to identify a region of the eutherian genome that is homologous to the region of the opossum genome containing *TRM*. In other words, and in contrast to the conventional TCR, the chromosomal region containing *TRM *is not well conserved in mammals.

Previously we reported that the TRM V gene segments appeared to be more similar to Ig V gene segments than that of TCR [14]. This conclusion was drawn from an analysis of a limited number of available marsupial TCR V gene segments at that time. Availability of the complete TCR genomic sequences described above allows us to further test this observation. *TRM *is organized in tandem clusters where complete clusters contain two classes of V segments: a single non-rearranged V gene segment (*TRMV*) and an unusual V gene segment which is already joined to D and J genes (*TRMVj*) in the germline DNA [14]. The opossum has six such complete clusters. The remaining two clusters are partial, lacking the *TRMV *and *TRMD *gene segments (Figure 8) [14].

To investigate the evolutionary history of the individual TRM clusters we compared the sequence and organization of the six complete clusters (clusters 1, 2, 3, 4, 5, and 7) and two partial clusters (clusters 6 and 8). These analyses revealed three classes of clusters based on gene content and nucleotide sequence identity. These three classes likely represent the most ancient duplications of TRM clusters. The lineages generated by these older duplications are represented by clusters 1, 2 and 3, respectively. Clusters 5 and 7 have similar gene content (three *TRMD *segments) and share greater nucleotide sequence identity to cluster 3 and represent more recent whole cluster duplications that would have followed the duplication of an additional D segment in this lineage [14]. Clusters 4, 6, and 8 contain two *TRMD *segments and share greatest nucleotide sequence identity to cluster 2, representing another round of more recent duplications. Cluster 1 is a third class unto itself based on not sharing significant sequence similarity to the others [14]. These results are consistent with the current complement of TRM clusters in opossum being the result of whole cluster duplications followed by divergence of each cluster. These duplication events have resulted in partial clusters cases and different numbers of clusters in different marsupial species; bandicoots for example appear to have only two TRM clusters [14,29].

**Figure 9:**
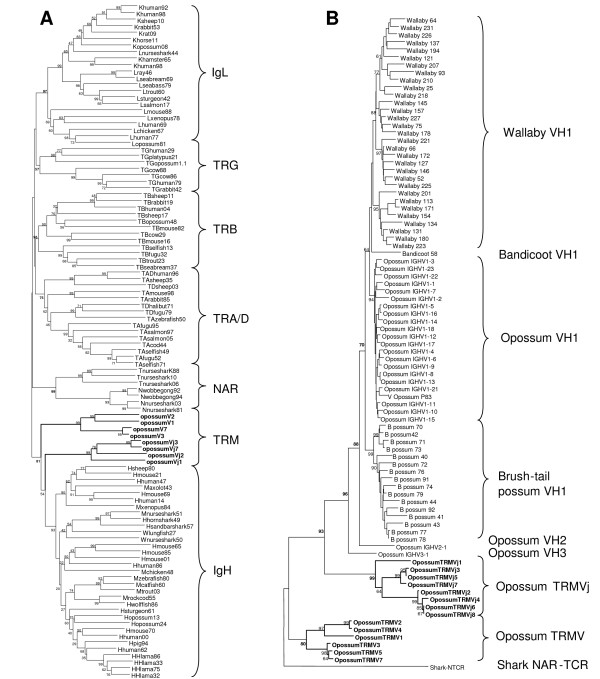
Phylogenetic analyses of TRMV to other V genes from TCR and Ig loci. **A**: The relationship of the V gene segments from *IgH, IgL, NAR, TRA, TRD, TRB, TRD *and *TRM*. Tree shown was generated using the neighbor joining method. Opossum *TRM *sequences are indicated in bold. Numbers at the nodes indicate the percent bootstrap values based on 1000 replicates. **B**: Phylogenetic tree showing the relationship of V gene segments from opossum TRM to IgH V gene segments from four distantly related marsupials. This analysis includes all the opossum IgH V genes which belong to three different subgroups (VH1–VH3). Available VH1 sequences from the brush-tail possum, tammar wallaby, Northern brown bandicoot and Virginia opossum were also used in the analysis. NAR-TCR was used as an outgroup. The tree was generated using the neighbor joining method with the Tajima-Nei model. Bootstrap values are indicated as in figure 9A.

Opossum *TRMV *and *TRMVj *each form distinct clades in a phylogenetic analysis and share only 38 to 43.5% nucleotide identity to each other and are, therefore, from distinct subgroups (Figure 9A) [14]. Now having the complete genomic sequence from the opossum, we wished to compare the TRMV genes with V genes from the conventional TCR and Ig loci. When *TRMV *and *TRMVj *are compared to all conventional opossum TCR V gene segments the similarity also remains low; nucleotide identity between TRM V genes and TRA, TRB, TRG, and TRD V genes ranges from 27 to 43%. In other words, the *TRA/D*, *TRB*, and *TRG *loci do not contain V segments from which either TRMV or TRMVj appear to have been derived. The greatest similarity for TRM V genes remains with the IgH V gene segments (VH) with nucleotide identity ranging from 34 to 51%. However, the clades of *TRMV *and *TRMVj *remain outside those containing *VH *genes from a variety of species (Figure 9A). In addition we compared the TRM V genes to all the germline VH in opossum and four other marsupial species (Tammar wallaby, Virginia opossum, Brush-tail possum, and Northern Brown bandicoot) and TRMV and IGHV continued to form distinct clades (Figure 9B). These results are consistent with a conclusion that, indeed, TRMV are most related to IGHV, but not sufficiently similar to extant marsupial IGHV to determine from which they might have been derived.

In addition to the six functional TRMV gene segments located within the *TRM *locus, there is a single TRMV orphon gene (TRMV-OR2) located on opossum chromosome 2 in a region containing a number of flanking sequences resembling long interspersed repeat elements (LINE)(data not shown). TRM is the only of the TCR loci in the opossum for which orphon V gene segments have been found. TRMV-OR2 appears to be non-functional and has no leader peptide, but it does contain a complete V gene segment and the recombination signal sequence (RSS). TRMV-OR2 is most similar to TRMV2 and TRMV4, sharing 96 and 95% nucleotide identity, respectively. The high degree of identity between these functional gene segments and the orphon is consistent with the latter being the result of a relatively recent duplication event that, based on the flanking LINE elements may have been due to transposition [39]. TRMV-OR2 provides additional evidence for the role of retroelements in both gene translocation in marsupials. A similar translocation was reported previously for opossum MHC class I genes, where two class I loci (UB and UC) had been translocated outside the MHC region. UB and UC are similarly tightly flanked by retroelements [40,41]. Furthermore, TRMV-OR2 may also provide an additional connection between retroelements and the evolution of TCR genes themselves in marsupials. TRMVj is a variable region gene segment that appears to have been generated by retro-transposition since it is lacking an intron that all V gene segments have and is already joined to the D and J gene segments in the germline [14]. Although highly speculative, it is possible that translocated orphons such as TRMV-OR2 may have contributed to the activation of local retroelement genes to allow their co-expression when the TRM locus is actively transcribed. This would have been a necessary step in the retro-translocation events that generated TRMVj.

### Is TRM present in lineages other than marsupials?

The availability of a large number of vertebrate genome sequences, with varying degrees of depth of sequence coverage, provided the opportunity to search for TRM or TRM-like genes in species other than marsupials. A search of the available genomes using the BLAST algorithm and opossum TRM sequences was unable to identify a homologue in any of the eutherian species available. This search included human, mouse, rabbit, dog, cat, cow, horse, hedgehog, elephant and armadillo. The rabbit, cat, hedgehog, elephant and armadillo are low coverage genome sequences ranging from 1.86× to 2×. While it is possible that in any given species the TRM locus was missed in the sequencing, it is unlikely that such a random gap would have been consistently present in all the eutherian genomes. Therefore we conclude that TRM is not likely present in any eutherian lineage. We also searched the current chicken and the anole lizard (*Anolis carolinensis*) genomes in a similar manner and were unable to detect clear TRM homologues. In the case of chicken and all the eutherian genomes, the TRD homologues were identifiable indicating that our search strategies are able to pick up this conventional TCR locus. Furthermore, we were able to identify a clear TRM homologue in the recently completed platypus genome sequence (Ornithorhynchus_anatinus-5.0) available at GenBank. Further characterization of the platypus TRM locus is ongoing and beyond the scope of this paper. Nonetheless identification of these genes in a monotreme, which are separated from marsupials and eutherians by 217 to 231 MY [15], further supports that our search strategies should be able to identify TRM homologues in other species. These results are also consistent with TRM being present early in the evolution of mammals and therefore likely lost in the eutherian lineage.

## Conclusion

First and foremost, we have described in detail the genomic content and complexity of the T cell receptor loci for the opossum *Monodelphis domestica*, the first such analysis available for a marsupial. The opossum is arguably the most extensively studied marsupial species and is used as a model of human disease and development. The opossum, for example, is one of the few mammalian model organisms that develop melanoma following exposure to ultraviolet radiation providing a cancer model [42]. Additionally, opossums are a natural host and a reservoir of the causative agent of Chagas disease, *Trypanosoma cruzi *[43]. Like all marsupials, opossums give birth to highly altricial young also providing a model for early immune as well as other anatomical system development. Further characterization of the immune system in the opossum, and T cell immunobiology in particular, is important for better understanding of these disease and developmental models. Complete characterization of the TCR genomics in this species is one step in that direction.

**Figure 10:**
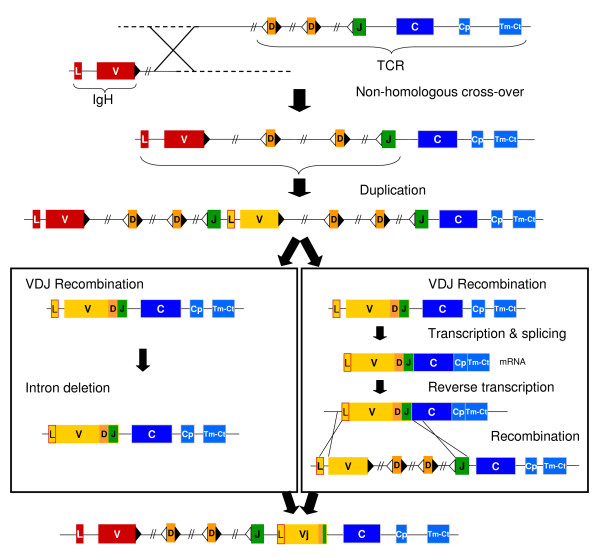
Diagram of possible scenarios for the origin of TRM. Non-homologous recombination between ancient IgH and TCR loci gave rise to a hybrid locus. A duplication event of part of the hybrid locus presumably created and additional set of V, D and J gene segments. Subsequently two possible alternatives are given for the origin of TRMVj. Left panel: A V(D)J recombination in germ cells followed by deletion of the intron located within the sequence encoding the leader peptide. Right panel: A V(D)J recombination event occurs in somatic cells, followed by transcription and splicing, resulting in a complete mRNA transcript. This mRNA was reverse transcribed to DNA by a reverse transcriptase and it was inserted back into the genome by homologous recombination. V, D and J gene segments and C regions are color-coded as in previous figures. TRMV and TRMVj are distinguished as red or yellow color, respectively. The exon encoding leader peptide is indicated with an L.

In spite of detailed analyses of the opossum conventional TCR loci, the origins of TRM remain enigmatic. The current evidence support the following conclusions and model for the origin of TRM: 1) There likely was a recombination or insertion event between an *IgH *and *TCR *locus (Figure 10); 2) The *TCR *locus involved was most likely *TRD *or a *TRD-like *based on sequence similarity [14]. Unfortunately the highly conserved and stable organization of the TRA/D region across birds and mammals does not provide clues as to how TRD might have participated in the origins of TRM; 3) The IGH-TCR hybrid formed likely underwent a whole or partial duplication event giving rise to multiple sets of V, D, and J elements one of which remained unrearranged in the germline, the other becoming germline joined either through direct RAG mediated V(D)J recombination in the germline (left-hand path in Figure 10) or through retrotransposition (right-hand path in Figure 10). Ongoing analyses of the TRM locus in the platypus may yield further insights into these possible scenarios for the origins of this unusual TCR chain.

## Methods

### Genome analyses

The opossum whole genome assembly MonDom5 was used in this study and it is available at GenBank under the accession number AAFR00000000[23]. The location for all TCR gene segments in MonDom5 is provided in Additional file 3. For comparative purposes the current genome assemblies from human (NCBI 36), mouse (NCBI m37), cow (Btau_3.1) and chicken (WASHUC2), rabbit (RABBIT), dog (CanFam2.0), cat (CAT), horse (*preEnsembl *EquCab2), hedgehog (eriEur1), elephant (BROAD E1), armadillo (ARMA) and the anole lizard (AnoCar1.0) were searched for any evidence of TRM. Genomes were analyzed using BLAST assembled genomes tools [23,35].

### RNA extraction

Opossum lymphoid tissues were collected and immediately processed to extract the RNA or stored in RNAlater^® ^(Ambion, Austin, TX) at 4°C for 24 hours and stored at -80°C for use later. Whole RNA extraction was performed using the Trizol RNA extraction protocol (Invitrogen, Carlsbad, CA). Tissue was homogenized in 1 ml of Trizol^® ^Reagent per 100 mg until the tissue was completely dispersed. Phase separation was done using 200 μl of chloroform per 1 ml of Trizol. RNA was precipitated with 500 μl of isopropanol per 1 ml of trizol, washed with 70% ethanol and resuspended in 50 to 100 μl of DEPC water. DNase treatment to remove contaminating DNA has been performed using Ambion's kit TURBO DNA-free (Ambion, Austin, TX). Each sample was quantified using the NanoDrop ND-1000 Spectrophotometer (NanoDrop Technologies, Wilmington, DE).

### Reverse transcription, PCR and sequencing

Reverse transcription-polymerase chain reactions (RT-PCR) were performed using GeneAmp RNA PCR Core Kit (Applied Biosystems, Foster City, CA). Amplifications of cDNAs were performed using AdvantageTM-HF 2 PCR (BD Biosciences, CLONTECH Laboratories, Palo Alto, California) with the conditions: 94°C for 1 minute, denaturation at 94°C for 30 seconds, annealing/extension according to the melting temperature of the primers, and a final extension period of 68°C for 5 minutes.

PCR products were cloned using TOPO TA Cloning^® ^Kit for sequencing (Invitrogen, Carlsbad, CA). Plasmids were sequenced using BigDye Terminator Cycle Sequencing Kit v3 (Applied Biosystems, Foster City, CA) in 10 μl reactions and analyzed on an ABI Prism 3100 DNA automated sequencer (PerkinElmer Life And Analytical Sciences Inc, Wellesley, MA). Analyses of chromatograms were done using the SequencherTM 4.6 program (Gene Codes Corporation, Ann Arbor, MI).

### Identification of TRV, TRD, TRJ, TRC genes

To determine the location, content and organization of the TCR genes, the whole opossum genome was searched using the BLAST algorithm. TRA, TRB, TRG, TRD and TRM were located using sequences previously isolated [28,29,38,14]. The V and J segments were located by similarity to corresponding segments from other species and by identifying the flanking conserved RSS.

Rapid amplification of 5' complementary DNA ends (5' RACE) performed on opossum thymus mRNA was used to identify novel, expressed V, D and J segments. In addition to using 5' RACE, PCR using primers that are specific for each V family were also used in RT-PCR to amplify cDNA containing VDJ recombinations that are underrepresented in the RACE PCR. Primers used to amplify the conventional TCR are complementary to the most conserved sequences of the V regions and have been paired with primers located on the C regions (Additional file 4). Sequences obtained by these means were compared with the whole opossum genome using the BLAST algorithm to identify novel TCR gene segments. This approach allowed the identification of gene segments in six possible reading frames, which also help to find gene segments located in the reverse orientation and D segments that may be used in multiple reading frames. The exon-intron organization of V regions was determined using sequences obtained by 5' RACE.

Determination of the exon-intron organization in the C regions was done using BLAST to compare available cDNA sequences that encode complete TCR chains to the opossum genomic sequences. Additionally, transcripts encoding the C terminal end of TCR were obtained by 3' RACE performed on thymus cDNA. Clones obtained by 3' RACE PCR were used to identify the CP, TM and CT and 3' untranslated regions (UTR) for each one of the TCR. cDNA made using the Oligo dT primer was used to perform 3' RACE PCR. The Oligo dT primer supplies a priming site for the GeneRacerTM 3' PCR primers. Sequences obtained by 3' RACE were aligned with the germline sequences to determine the location, intron-exon boundaries and splice sites of these exons.

### Nomenclature

Opossum gene segments were named following the IMGT nomenclature established for human and mouse [44]. TRV segments were numbered according to their location, from the 5' to 3' end of the locus. TRAJ segments were numbered from 3' to 5' according to a nomenclature proposed by Koop *et al*. [45] and followed by IMGT. The TRBD, TRBJ and TRBC found in four cassettes in the opossum are numbered according to the cassette to which they belong and the position of the cassette from 5' to 3' in the locus.

### Phylogenetic analyses

Nucleotide sequences that encode from FR1 through FR3 of the V regions identified from each one of the opossum TCR loci were compared to TCR V sequences from other species retrieved from GenBank. The sequences were aligned using ClustalX [46] and BioEdit [47] programs. Phylogenetic analyses were performed using MEGA version 3.0 [48] for the distance methods (neighbor joining and minimum evolution). Confidence values were obtained from bootstrap analyses using 1000 replications.

The accession numbers for sequences used in the phylogenetic analyses are:

#### Figure 2

**Human**: A8.4: D13077; A3: X57534; A16: DQ097913; A9.2: X57531; A40: DQ097942; A10: DQ097904; A27: DQ341447; A25: DQ097923; A35: DQ097935; A22: DQ097920; A20: X70305; A34: DQ097934; A30: X58768; A41: DQ097943; A36/DV7: X61070; A17: DQ097914; A6: X58747; A24: M17661; A21: U50404; A23/DV6: D13071; A29/DV5: M17664; A1.1: X04939; A26.1: L06886; A4: M17663; A38-2/DV8: D13074; A14: S51029; D3: M23326; D1: AY357942; D2: S24406; A19: Z46641; A2: DQ097917. **Mouse**: A9: M33586; A17: X60319; A12.3: M38680; A6: M34200; A11: DQ340292; A13: M38102; A4: L47342; A7: X56719; A14.3: L77149; A5.4: M38681; A10: X57397; A3.1: X02967; A1: M22604; A2: X03760; A21/DV12: M94080; A15-2/DV6-2: M37599; D5: X12729; D4: M23545; TRADV16D: M16118; D2.2: M37280. **Rabbit**: D4: 38120; 885: M12885; D1: D26555; D5: D38121. **Sheep**: A622: M55622; D1S1: Z12989; D6: AJ005908; D7: AJ809501; D4: AJ005906; D2: AJ005904; D5: Z12995; D3S2: Z12996; A35: U78035. **Cow**: 014: D90014; 013: D90013; 015: D90015; 011: D90011; 012: D90012; 017: D90017; D113: D16113; 016: D90016; D116: D16116. **Chicken**: 611: GDU04611; 612: GDU04612; 613: GDU04613.

#### Figure 5

**Human**: B11.3: X58797; B7.2: U07975; B12.1: X07224; B14: X06154; B17: U03115; B2: M64351; B27: U66061; B25.1: L27610; B13: U03115; B4.3: X58812; B21.1: L27608; B23: L27614; B5: X61439; B9: M27380; B3.1: U07977; B15: U03115; B24.1: L27612; B10.3: U17047; B28: U08314; B6.9: X61447; B19: U48260; B30: L06893; B20.1: X72719; B29.1: M13847. **Mouse**: B14: AE000664; B16: L29434; B3: AE000663; B21: X16691; B26: K02548; B23: X59150; B24: M61184; B12: M30881; B2: AE000663; B4: X56725; B17: AE000664; B29: X00696; B10: X16694; B13.3: M15616; B19: AJ249821; B31: X03277; B30: X16695; B20: M11859; B1: X01642. **Rabbit**: B8: BAA04245; B11: BAA04248; B9: BAA04246; B7S1: BAA04241; B1: AAA31472; B5: BAA04239; B10: BAA04247; B2: M13895; B6: BAA04240. **Sheep**: B6S1: AAB88431; B8S1: AAB88433; B10S1: AAB88434; B1S5: AAB88425; B7S1: AAB88432; B22S1: AAB88440; B3S1: AAB88427; B13S1: PQ0068; B15S1: AAB88438; B12S1: AAB88435; B17S1: AAB88439; B2S1: AAB88426; B4S1: AAB88430. **Cow**: B126: PQ0062; B129: PQ0065; B125: PQ0061; B122: JQ0473; B123: PQ0060; B124: PQ0059. **Chicken**: B1S1: B36198; B2S2: AAA62753.

#### Figure 7

**Human**: G2: M13429; G3: S60779; G4: S60780; G2: M27335. **Mouse**: G3: AF037352; G6: M13338; G4: M13336; G7: AF037352; G1: Z22847. **Rabbit**: G1S2: D38137; G1S3: D38138; G1S1: D38135; G1S4: D38139; G2S1: D38142. **Sheep**: G6S1: Z13007; G2S1: Z12999; G2S2: Z13000; G2S3: Z13001; G2S4: Z13002; G5S1: Z13005; G5S2: Z13006; G1S1: Z12998; G3S1: Z13003; G4S1: Z13004. **Cow**: G6: AY560834; G1S1: D16119; G1S3: D16131; G5S11: D16126; G5S7: D16130; G5S6: D16129; G5S16: D16133; G3S1: U73186; G3S2: U73187; G4S1: U73188. **Platypus**: V3.1: AAY82120; V3.2: AAY82094; V1.1: AAY82100; V1.2: AAY82093; V1.3: AAY82109; V2.2: AAY82110; V2.3: AAY82114; V2.1: AAY82119. **Chicken**: G3S8: U78235; G3S4: U78231; G3S3: U78230; G2S7: U78225; G2S8: U78226; G2S9: U78227; G1S4: U78212; G1S5: U78213; G1S3: U78210; G1S8: U78216.

#### Figure 9A

**IgH**: Hsheep80: U80145; Hmouse21: M27021; Hhuman47: X64147; Maxolot43: L20243; Hmouse69: K01569; Hhuman14: X05714; Mxenopus84: M20484; Hmouse70: M21470; Hhuman00: DQ454900; HHlama75: AY544575; HHlama33: AY342133; HHlama32: AJ629032; HHlama86: AF441486; Hpig94: U15194; Hhuman62: U80162; Hopossum13: AF012113; Hopossum24: AF012124; Hhuman86: M99686; Hmouse65: M25465; Hmouse85: M31285; Hmouse01: X03301; Mchicken48: M30348; Mzebrafish80: AF281480; Mcatfish60: DQ230560; Mtrout03: DQ831803; Mrockcod55: AF303555; Hwolffish86: AY188786; Hsturgeon61: DQ257661; Wnurseshark50: U51450; Wlungfish27: AF437727; Mnurseshark51: M92851; Hhornshark49: X13449; Hsandbarshark57: AY548357. **NAR**-**TCR**: TnarsharK88: DQ022688; Tnarshark10: DQ022710; Tnarshark06: DQ022706. **NAR**: Nwobbegong92: AF336092; Nwobbegong94: AF336094; Nnurseshar03: AF447103; Nnurseshar81: AY114781. **IgL**: Khuman92: Y08392; Khuman98: DQ915098; Ksheep10: X54110; Krabbit53: AF211353; Krat09: U39609; Khorse11: X75611; Kopossum08: AY074408; Lnurseshark44: GCU15144; Khamster65: U17165; Khuman98: AY320598; Lmouse88: DQ986488; Lray46: AB062646; Lseabream69: EF555069; Lsturgeon42: AJ387842; Lsalmon17: AF273017; Lseabass79: AJ291779; Ltrout60: X65260; Lhuman77: AF216777; Lopossum81: AF049781; Lchicken67: S65967; Lxenopus78: L76578; Lhuman69: Z73669. **TRG**: TGhuman29: M13429; TGplatypus21: DQ011321; TAmouse98: M34198; TArabbit85: M12885; TGhuman79: S60779; TGcow86: U73186; TGrabbit42: D38142; TGcow88: U73188. **TRA/D**: TAsalmon97: EF467297; TAsalmon05: EF466505; TAfugu52: AY198352; TAselfish71: AY198371; TAselfish49: AY198349; TAcod44: AJ133844; TAfugu95: AB222395; TDhalibut71: AB076071; TDfugu79: AB222479; TAzebrafish50: AL592550; TADhuman96: Z14996; TAsheep35: U78035; TDsheep03: AJ005903. **TRB**: TBtrout23: OSU18123; TBseabream37: AM490437; TBfugu32: AB222432; TBselfish13: AF324813; TBopossum8.1: XM_001363148; TBmouse82: DQ983582; TBsheep11: AF030011; TBrabbit19: D17419; TBhuman04: M27904; TBsheep17: AF030017; TBcow29: D90129; TBmouse16: M15616.

#### Figure 9B

**Gray short-tailed opossum**: IGHV1-1: AAC48826; IGHV1-6: AAC48820; IGHV1-11: AAC48816; IGHV1-16: AAC48836; IGHV1-23: AAC48846; IGHV2-1: AAC48849. Remaining germline sequences are available at AAFR00000000. **Brush-tail possum**: 70: AAL87470; 71: AAL87471; 72: AAL87472; 73: AAL87473; 74: AAL87474; 76: AAL87476; 77: AAL87477; 78: AAL87478; 79: AAL87479; 91: AAD41691; 92: AAD41692; 41: AAT40441; 40: AAT40440; 44: AAT40444; 43: AAT40443; 42: AAT40442. **Virginia opossum**: P83 is an unpublished sequence kindly provided by Dr. R. Riblet. **Tammar Wallaby**: sequences are unpublished but available upon request. **Northern Brown Bandicoot**: Bandicoot58: AY586158. **Shark **NTCR: AAY98815.

### Dot plot analyses

Comparisons of the genomic sequence were performed using the program Spin from the Staden Package [49]. Dot matrix plots were generated to determine degrees of similarity among cassettes for the TRB locus. The sequence analyzed includes the four TRB cassettes with coding and non-coding sequence from 3kb upstream of the most 5' TRBD (*TRBD1*) segment to the most 3' TRBC (*TRBC4*) segment, which comprises 51 kb. Although not shown, similar comparisons were performed for the other TCR loci.

## Abbreviations

TCR: T cell receptor. TRA: T cell receptor alpha. TRB: T cell receptor beta. TRG: T cell receptor gamma. TRD: T cell receptor delta. TRM: T cell receptor mu. Ig: immunoglobulin. V: variable gene segment. D: Diversity gene segment. J: Joining gene segment. C: constant region. Tm: transmembrane region. Cp: connecting peptide. Ct: cytoplasmic region. RSS: recombination signal sequence. MHC: major histocompatibility complex.

## Authors' contributions

ZEP, MLB, and RDM conceived of the study, participated in its design, and drafted the manuscript. ZEP, JH, AML, JT, and AS generated the data and performed the sequencing reactions. ZEP performed the data analyses. All authors have read and approved the final manuscript.

## Supplementary Material

Additional file 1 Opossum TCR syntenic genes and their corresponding location on chromosomes of several species. this table contains the opossum TCR syntenic genes and their corresponding location on chromosomes of several species. Click here for file

Additional file 2 Dot plot analyses of opossum TRB cassettes. The dot-matrix analysis corresponds to the comparison of 51 Kb region containing the four TRB D-J-C cassettes aligned to itself. Click here for file

Additional file 3 Location of opossum TRA/D, TRB, TRG and TRM gene segments. contains four tables (A-D) with the location of opossum TRA/TRD, TRB, TRG and TRM gene segments in the MonDom5 assembly. Click here for file

Additional file 4 List of primers used to amplify opossum TCR. this table contains the sequences of forward and reverse primers used to amplify opossum TCR. Click here for file

## References

[B1] Kronenberg M, Siu G, Hood LE, Shastri N (1986). The molecular genetics of the T cell antigen receptor and T cell antigen recognition. Ann Rev Immunol.

[B2] Clevers H, Alarcon B, Wileman T, Terhorst C (1988). The T Cell Receptor/CD3 Complex: A Dynamic Protein Ensemble. Ann Rev Immunol.

[B3] Brenner MB, McLean J, Dialynas DP, Strominger JL, Smith JA, Owen FL, Seidman JG, Rosen SF, Krangel MS (1986). Identification of a putative second T-cell receptor. Nature.

[B4] Hayday AC (2000). γδ cells: a right time and a right place for a conserved third way of protection. Ann Rev Immunol.

[B5] Davis MM, Chein YH (2003). Fundamental Immunology.

[B6] Marchalonis JJ, Schluter SF (1989). Evolution of variable and constant domains and joining segments of rearranging immunoglobulins. FASEB J.

[B7] Marchalonis JJ, Bernstein RM, Shen SX, Schluter SF (1996). Emergence of the immunoglobulin family: conservation in protein sequence and plasticity in gene organization. Glycobiology.

[B8] Rast JP, Anderson MK, Strong SJ, Luer C, Litman RT, Litman GW (1997). α, β, γ and δT cell antigen receptor genes arose early in vertebrate phylogeny. Immunity.

[B9] Flajnik MF (2005). The last flag unfurled? A new immunoglobulin isotype in fish expressed in early development. Nat Immunol.

[B10] Hsu E, Pulhman N, Rumfelt LL, Flajnik MF (2006). The plasticity of immunoglobulin gene systems in evolution. Immunol Rev.

[B11] Flajnik MF (2002). Comparative analyses of immunoglobulin genes: surprises and portents. Nat Rev Immunol.

[B12] Richards MH, Nelson JL (2000). The evolution of vertebrate antigen receptors: a phylogenetic approach. Mol Biol Evol.

[B13] Criscitiello MF, Saltis M, Flajnik MF (2006). An evolutionarily mobile antigen receptor variable region gene: Doubly rearranging NAR-TcR genes in sharks. Proc Natl Acad Sci USA.

[B14] Parra ZE, Baker ML, Schwarz R, Deakin J, Lindblad-Toh K, Miller RD (2007). A unique T cell receptor discovered in marsupials. Proc Natl Acad Sci USA.

[B15] Rheede TV, Bastiaans T, Boone DN, Hedges SB, De-Jong WW, Madsen O (2006). The Platypus Is in Its Place: Nuclear Genes and Indels Confirm the Sister Group Relation of Monotremes and Therians. Mol Biol Evol.

[B16] Mikkelsen TJ, Wakefield MJ, Aken B, Amemiya CT, Chang JL, Duke S, Garber M, Gentles AJ, Goodstadt L, Heger A, Jurka J, Kamal M, Mauceli E, Searle SMJ, Sharpe T, Baker ML, Batzer MA, Venos PV, Belov K, Clamp M, Cook A, Cuff J, Das R, Davidow L, Deakin JE, Fazzari MJ, Glass JL, Grabherr M, Greally JM, Gu W, Hore TA, Huttley GA, Jirtle RL, Koina E, Lee JT, Mahony S, Marra MA, Miller RD, Nicholls RD, Oda M, Papemfuss AT, Parra ZE, Pollock DD, Ray DA, Schein JE, Speed TP, Thompson K, VandeBerg JL, Wade CM, Walker JA, Waters PD, Webber C, Weidman JR, Xie X, Zody MC, Marshall-Graves JA, Ponting CP, Breen M, Samollow PB, Lander ES, Lindblad-Toh K, Broad Institute Genome Sequencing Platform, Broad Institute Whole Genome Assembly Team (2007). Genome of the marsupial *Monodelphis domestica *reveals adaptive turnover of coding and non-coding sequences. Nature.

[B17] Deakin JE, Parra ZE, Graves JAM, Miller RD (2006). Physical Mapping of T cell receptor loci (TRA, TRB, TRD and TRG) in the opossum (*Monodelphis domestica*). Cytogenet Genome Res.

[B18] Satyanarayana K, Hata S, Devlin P, Roncarolo MG, De Vries JE, Spits H, Strominger JL, Krangel MS (1988). Genomic organization of the human T-cell antigen-receptor α/δ locus. Proc Natl Acad Sci USA.

[B19] Chien YH, Iwashima M, Kaplan KB, Elliot JF, Davis MM (1987). A new T-cell receptor gene located within the alpha locus and expressed early in T-cell differentiation. Nature.

[B20] Kubota T, Wang JY, Gobel TWF, Hockett RD, Cooper MD, Chen CH (1999). Characterization of an avian (*Gallus gallus domesticus*) TCR αδ locus. J Immunol.

[B21] Giudicelli V, Chaume D, Lefranc MP (2005). IMGT/GENE-DB: a comprehensive database for human and mouse immunoglobulin and T cell receptor genes. Nucleic Acids Res.

[B22] Glusman G, Rowen L, Lee I, Boysen C, Roach JC, Smith AFA, Wang K, Koop BF, Hood L (2001). Comparative genomics of the human and mouse T cell receptor loci. Immunity.

[B23] ENSEMBL. http://www.ensembl.org.

[B24] Hedges SB, Parker PH, Sibley CG, Kumar S (1996). Continental breakup and the ordinal diversification of birds and mammals. Nature.

[B25] Hubbard GB, Saphire DG, Hackleman SM, Silva MV, Vanderberg JL, Stone WH (1991). Ontongeny of the thymus gland of a marsupial (*Monodelphis domestica*) Laboratory animal. Science.

[B26] Nei M, Gu X, Sitnikova T (1997). Evolution by the birth and death process in multigene families of the vertebrate immune system. Proc Natl Acad Sci USA.

[B27] Su C, Jakobsen I, Gu X, Nei M (1999). Diversity and evolution of T-cell receptor variable region genes in mammals and birds. Immunogenetics.

[B28] Baker ML, Rosenberg GH, Zuccolotto P, Harrison GA, Deane EM, Miller RD (2001). Further characterization of T cell receptor chains of marsupials. Dev Comp Immunol.

[B29] Baker ML, Osterman AK, Brumburgh S (2005). Divergent T cell receptor delta chains from marsupials. Immunogenetics.

[B30] Call ME, Wucherpfennig KW (2005). The T cell receptor: critical role of the membrane environment in receptor assembly and function. Ann Rev Immunol.

[B31] Rowen L, Koop BF, Hood L (1996). The complete 685-kilobase DNA sequence of the Human βT cell receptor locus. Science.

[B32] Lefranc MP, Lefranc G (2001). The T Cell Receptor FactsBook.

[B33] Vernooij BT, Lenstra JA, Wang K, Hood L (1993). Organization of the murine T-cell receptor gamma locus. Genomics.

[B34] Lefranc MP, Rabbitts TH (1985). Two tandemly organized human genes encoding the T-cell gamma constant region sequences show multiple rearrangement in different T-cell types. Nature.

[B35] NCBI. http://www.ncbi.nlm.nih.gov/BLAST/.

[B36] Miccoli MC, Antonacci R, Vaccarelli G, Lanave C, Massari S, Cribiu EP, Ciccarese S (2003). Evolution of TRG clusters in cattle and sheep genomes as drawn from the structural analysis of the ovine TRG2@ locus. J Mol Evol.

[B37] Conrad ML, Mawer MA, Lefranc MP, McKinnell L, Whitehead J, Davis SK, Pettman R, Koop BF (2007). The genomic sequence of the bovine T cell receptor gamma TRG loci and localization of the TRGC5 cassette. Vet Immunol Immunopathol.

[B38] Parra ZE, Arnold T, Nowak MA, Hellman L, Miller RD (2006). TCR gamma chain diversity in the spleen of the duckbill platypus (*Ornithorhynchus anatinus*). Dev Comp Immunol.

[B39] Childs G, Maxson R, Cohn RH, Kedes L (1981). Orphons: dispersed genetic elements derived from tandem repetitive genes of eukaryotes. Cell.

[B40] Miska KB, Wright AM, Lundgren R, Sasaki-McClees R, Osterman AK, Gale JM, Miller RD (2004). Analysis of a marsupial MHC region containing two recently duplicated class I loci. Mamm Genome.

[B41] Belov K, Deakin JE, Papenfuss AT, Baker ML, Melman SD, Siddle HV, Gouin N, Goode DL, Sargeant TJ, Robinson MD, Wakefield MJ, Mahony S, Cross JG, Benos PV, Samollow PB, Spedd TP, Graves JA, Miller RD (2006). Reconstructing an ancestral mammalian immune supercomplex from a marsupial major histocompatibility complex. PLOS Biol.

[B42] Ley RD, Reeve VE, Kusewitt DF (2000). Photobiology of *Monodelphis domestica*. Dev Comp Immunol.

[B43] Teixeira AR, Monteiro PS, Rebelo JM, Arganaraz ER, Vieira D, Lauria-Pires L, Nascimento R, Vexenat CA, Silva AR, Ault SK, Costa JM (2001). Emerging Chagas Disease: Trophic Network and Cycle of Transmission of *Trypanosoma cruzi *from Palm Trees in the Amazon. Emerg Infect Dis.

[B44] IMGT. http://imgt.cines.fr/.

[B45] Koop BF, Rowen L, Wang K, Kuo CL, Seto D, Lenstra JA, Howard S, Shan W, Deahpande P, Hood L (1994). The Human T-cell receptor TCRAC/TCRDC (Cα/Cδ) region: Organization, sequence, and evolution of 976 kb of DNA. Genomics.

[B46] Thompson JD, Gibson TJ, Plewniak F, Jeanmougin F, Higgins DG (1997). The ClustalX windows interface: flexible strategies for multiple sequence alignment aided by quality analysis tools. Nucleic Acids Res.

[B47] Hall TA (1999). BioEdit: a user-friendly biological sequence alignment editor and analysis program for Windows 95/98/NT. Nucleic Acids Symp Ser.

[B48] Kumar S, Tamura K, Nei M (2004). MEGA3: Integrated software for Molecular Evolutionary Genetics Analysis and sequence alignment. Brief Bioinform.

[B49] Staden package. http://staden.sourceforge.net/.

