# Supplementary material for: Comparative genomic analysis and evolution of the T cell receptor loci in the opossum Monodelphis domestica
Source: BMC Genomics. 2008 Feb 29;9:111. doi: 10.1186/1471-2164-9-111 (PMC2275272; doi:10.1186/1471-2164-9-111)
Supplement: Additional file 1 — Opossum TCR syntenic genes and their corresponding location on chromosomes of several species. this table contains the opossum TCR syntenic genes and their corresponding location on chromosomes of several species. [file 1471-2164-9-111-S1.pdf]

Additional data file 1. Opossum TCR syntenic genes and their corresponding location on chromosomes of several species.

	**Human**	**Mouse**	**Cow**	**Chicken**
**TRA/D**	**14**	**14**	**10**	**27**
METTL3	14	14	10	NF
SALL2	14	14	10	NF
OR	14	14	10	NF
DAD-1	14	14	10	27
ABHD4	14	14	10	NF
**TRB**	**7**	**6**	**4**	**1**
TRY	7	6	4	1
CYCLIN A2	4	3	4	4
EPBH6	7	6	4	1
KELL	7	6	4	1
OTRPC3	7	6	4	1
DBHL	7	6	4	NF
**TRG**	**7**	**13**	**4**	**2**
AMPH	7	13	4	2
STARD3nl	7	13	4	2
**TRM**	**NF**	**NF**	**NF**	**NF**
ZNF3	7	5	25	1
PCIF-1	17	11	19	7
MOG	6	17	23	NF

NF: Not found in current assemblies
