# Supplementary material for: Comparative genomic analysis and evolution of the T cell receptor loci in the opossum Monodelphis domestica
Source: BMC Genomics. 2008 Feb 29;9:111. doi: 10.1186/1471-2164-9-111 (PMC2275272; doi:10.1186/1471-2164-9-111)
Supplement: Additional file 2 — Dot plot analyses of opossum TRB cassettes. The dot-matrix analysis corresponds to the comparison of 51 Kb region containing the four TRB D-J-C cassettes aligned to itself. [file 1471-2164-9-111-S2.pdf]

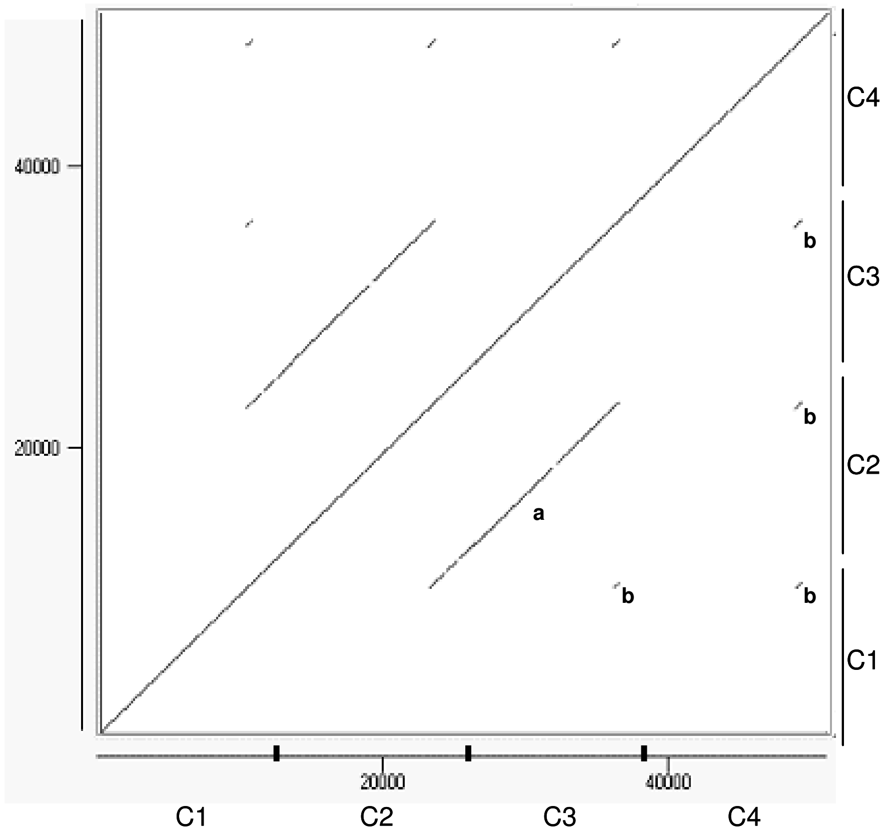

Additional Data File 2. Dot plot analyses of opossum TRB cassettes (C). The dot-matrix analysis corresponds to the comparison of the 51 kb region containing the four TRB D-J-C cassettes aligned to itself. Diagonal lines indicate internal repeats (except the main diagonal line) on the 51 kb analyzed. **a:** Coding and non-coding sequence similarity between TRBC2 and TRBC3 cassettes. **b:** Sequence similarities between exon 1 of TRBC regions.
