# Supplementary material for: Comparative genomic analysis and evolution of the T cell receptor loci in the opossum Monodelphis domestica
Source: BMC Genomics. 2008 Feb 29;9:111. doi: 10.1186/1471-2164-9-111 (PMC2275272; doi:10.1186/1471-2164-9-111)
Supplement: Additional file 3 — Location of opossum TRA/D, TRB, TRG and TRM gene segments. contains four tables (A-D) with the location of opossum TRA/TRD, TRB, TRG and TRM gene segments in the MonDom5 assembly. [file 1471-2164-9-111-S3.pdf]

Additional data file 3A. Location of opossum TRA and TRD gene segments†.

	FR1	RSS		FR1	RSS
**TRAV1**	1.170335765	1.170336039	**TRAV26**	1.170980543	1.170980820
**TRAV2.1**	1.170467363	1.170467645	**TRAV27**	1.171002055	1.171002328
**TRAV3**	1.170526288	1.170526564	**TRAV28**	1.171027496	1.171027772
**TRAV4ψ**	1.170536576	1.170536868	**TRAV29.1**	1.171035714	1.171035997
**TRAV5**	1.170551083	1.170551362	**TRAV29.2**	1.171038664	1.171038947
**TRAV6**	1.170565257	1.170565537	**TRAV29.3**	1.171051793	1.171052076
**TRAV7**	1.170570283	1.170570572	**TRDV1**	1.171063965	1.171064253
**TRAV8**	1.170580077	1.170580353	**TRAV29.4**	1.171072970	1.171073253
**TRAV9ψ**	1.170594428	1.170594725	**TRAV30**	1.171081236	1.171081512
**TRAV10**	1.170603678	1.170603957	**TRAV31**	1.171091982	1.171092270
**TRAV11ψ**	1.170612573	1.170612867	**TRAV32**	1.171098462	1.171098732
**TRAV12.1**	1.170631865	1.170632142	**TRAV33**	1.171110957	1.171111236
**TRAV12.2ψ**	1.170655644	1.170655934	**TRAV34**	1.171131484	1.171131760
**TRAV12.3**	1.170659620	1.170659899	**TRAV35**	1.171161426	1.171161696
**TRAV12.4ψ**	1.170662774	1.170663071	**TRAV36**	1.171163817	1.171164087
**TRAV12.5**	1.170690630	1.170690909	**TRAV37.1**	1.171187291	1.171187561
**TRAV12.6**	1.170694290	1.170694569	**TRAV38.1**	1.171195327	1.171195596
**TRAV12.7ψ**	1.170712407	1.170712698	**TRAV37.2**	1.171221691	1.171221961
**TRAV13ψ**	1.170726196	1.170726486	**TRAV38.2**	1.171225336	1.171225609
**TRAV14ψ**	1.170734085	1.170734354	**TRAV38.3**	1.171229567	1.171229840
**TRAV15ψ**	1.170741534	1.170741821	**TRAV38.4**	1.171246367	1.171246640
**TRAV16ψ**	1.170754219	1.170754530	**TRAV38.5**	1.171254122	1.171254395
**TRAV17.1**	1.170764244	1.170764515	**TRAV38.6**	1.171259736	1.171260009
**TRAV17.2ψ**	1.170785590	1.170785873	**TRAV39**	1.171264424	1.171264697
**TRAV18.1**	1.170796813	1.170797092	**TRAV40.1**	1.171287280	1.171287553
**TRAV19ψ**	1.170811615	1.170811892	**TRAV41.1**	1.171292594	1.171292864
**TRAV17.3**	1.170825363	1.170825634	**TRAV40.2**	1.171299775	1.171300048
**TRAV18.2**	1.170847937	1.170848216	**TRAV41.2**	1.171307433	1.171307703
**TRAV20**	1.170859120	1.170859409	**TRAV40.3**	1.171311478	1.171311751
**TRAV21**	1.170871041	1.170871316	**TRAV41.3**	1.171318193	1.171318466
**TRAV22**	1.170880386	1.170880663	**TRAV41.4**	1.171327657	1.171327930
**TRAV23**	1.170906947	1.170907224	**TRDV2**	1.171400567	1.171400854
**TRAV24**	1.170920360	1.170920622	**TRDV3**	1.171424064	1.171424350
**TRAV2.2**	1.170935383	1.170935665	**TRDV4ψ**	1.171444222	1.171444506
**TRAV2.3**	1.170938391	1.170938673	**TRDV5ψ**	1.171458241	1.171458383
**TRAV2.4**	1.170946870	1.170947152	**TRDV6**	1.171516536	1.171516242
**TRAV2.5**	1.170950407	1.170950700			
**TRAV25**	1.170975968	1.170976247			

Additional data file 3A. Location of opossum TRA and TRD gene segments†.

	RSS	3' Splice site		RSS	3' Splice site
**TRAJ1**	1.171624144	1.171624209	**TRAJ39**	1.171560884	1.171560947
**TRAJ2**	1.171621316	1.171621377	**TRAJ40**	1.171558092	1.171558150
**TRAJ3**	1.171618883	1.171618942	**TRAJ41**	1.171557219	1.171557281
**TRAJ4**	1.171616645	1.171616702	**TRAJ42**	1.171553702	1.171553764
**TRAJ5**	1.171614765	1.171614825	**TRAJ43**	1.171551960	1.171552026
**TRAJ6**	1.171611806	1.171611865	**TRAJ44**	1.171551108	1.171551168
**TRAJ7**	1.171610105	1.171610167	**TRAJ45**	1.171548555	1.171548620
**TRAJ8**	1.171607962	1.171608027	**TRAJ46**	1.171547901	1.171547966
**TRAJ9**	1.171607046	1.171607105	**TRAJ47**	1.171547329	1.171547386
**TRAJ10**	1.171606681	1.171606743	**TRAJ48**	1.171545900	1.171545956
**TRAJ11**	1.171605340	1.171605398	**TRAJ49**	1.171544979	1.171545042
**TRAJ12**	1.171603483	1.171603542	**TRAJ50**	1.171543006	1.171543068
**TRAJ13**	1.171602803	1.171602861	**TRAJ51**	1.171541297	1.171541356
**TRAJ14**	1.171600608	1.171600670	**TRAJ52**	1.171540591	1.171540649
**TRAJ15**	1.171598526	1.171598582	**TRAJ53**	1.171539502	1.171539560
**TRAJ16**	1.171596687	1.171596742		**5' RSS**	**3' RSS**
**TRAJ17**	1.171594781	1.171594842	**TRDD1**	1.171465157	1.171465172
**TRAJ18**	1.171593777	1.171593839	**TRDD2**	1.171481001	1.171481014
**TRAJ19**	1.171592376	1.171592435		**RSS**	**3' Splice site**
**TRAJ20**	1.171589459	1.171589517	**TRDJ1**	1.171481982	1.171482036
**TRAJ21**	1.171588810	1.171588872	**TRDJ2**	1.171487815	1.171487863
**TRAJ22**	1.171587455	1.171587511	**TRDJ3**	1.171490531	1.171490581
**TRAJ23**	1.171586283	1.171586339	**TRDJ4ψ**	1.171493331	1.171493382
**TRAJ24**	1.171583500	1.171583563	**TRDJ5**	1.171494186	1.171494239
**TRAJ25**	1.171582540	1.171582596	**TRDJ6**	1.171497391	1.171497449
**TRAJ26**	1.171581875	1.171581937			
**TRAJ27**	1.171580244	1.171580303		**5' Splice site**	**3' Splice site**
**TRAJ28**	1.171577416	1.171577458	**TRAC**	1.171630034	1.171630310
**TRAJ29**	1.171576717	1.171576779	**TRDC**	1.171502250	1.171502539
**TRAJ30**	1.171575553	1.171575618			
**TRAJ31**	1.171573466	1.171573526			
**TRAJ32**	1.171570739	1.171570798			
**TRAJ33**	1.171569626	1.171569688			
**TRAJ34**	1.171568881	1.171568944			
**TRAJ35**	1.171567118	1.171567174			
**TRAJ36**	1.171565234	1.171565296			
**TRAJ37**	1.171563013	1.171563073			
**TRAJ38**	1.171561293	1.171561355			

Additional data file 3B. Location of opossum TRB gene segments†.

	FR1	RSS		RSS	3' Splice site
**TRBV1**	8.204993821	8.204994110	**TRBJ1.1**	8.205327531	8.205327579
**TRBV2ψ**	8.204997589	8.204997849	**TRBJ1.2**	8.205327665	8.205327712
**TRBV3**	8.205004642	8.205004925	**TRBJ1.3**	8.205328456	8.205328505
**TRBV4**	8.205024053	8.205024342	**TRBJ1.4**	8.205329278	8.205329326
**TRBV5ψ**	8.205031771	8.205032065	**TRBJ1.5**	8.205330209	8.205330258
**TRBV6.1**	8.205042403	8.205042689	**TRBJ2.1**	8.205342560	8.205342606
**TRBV6.2**	8.205045926	8.205046212	**TRBJ2.2**	8.205342841	8.205342891
**TRBV6.3**	8.205047991	8.205048277	**TRBJ2.3**	8.205343102	8.205343148
**TRBV7ψ**	8.205051494	8.205051783	**TRBJ2.4**	8.205343276	8.205343329
**TRBV8.1**	8.205073658	8.205073944	**TRBJ3.1**	8.205355423	8.205355469
**TRBV8.2**	8.205077939	8.205078225	**TRBJ3.2**	8.205355704	8.205355754
**TRBV9**	8.205100683	8.205100972	**TRBJ3.3**	8.205355960	8.205356006
**TRBV10ψ**	8.205116394	8.205116679	**TRBJ3.4**	8.205356134	8.205356187
**TRBV11.1**	8.205125777	8.205126064	**TRBJ4.1**	8.205365746	8.205365792
**TRBV12.1ψ**	8.205126691	8.205126989	**TRBJ4.2**	8.205366024	8.205366074
**TRBV13.1**	8.205132530	8.205132819	**TRBJ4.3**	8.205366239	8.205366287
**TRBV11.2**	8.205143839	8.205144126	**TRBJ4.4**	8.205366414	8.205366463
**TRBV12.2**	8.205145592	8.205145881	**TRBJ4.5**	8.205366749	8.205366797
**TRBV14.1ψ**	8.205150669	8.205150966		**5' RSS**	**3' RSS**
**TRBV14.2ψ**	8.205154580	8.205154869	**TRBD1**	8.205326651	8.205326663
**TRBV13.2**	8.205158158	8.205158447	**TRBD2**	8.205341965	8.205341982
**TRBV15ψ**	8.205169126	8.205169415	**TRBD3**	8.205354831	8.205354846
**TRBV13.3**	8.205172206	8.205172492	**TRBD4**	8.205365180	8.205365197
**TRBV16**	8.205174882	8.205175174			
**TRBV17**	8.205181905	8.205182179			
**TRBV18ψ**	8.205189576	8.205189870		**5' Splice site**	**3' Splice site**
**TRBV19**	8.205200901	8.205201186	**TRBC1**	8.205333468	8.205333865
**TRBV20**	8.205205494	8.205205775	**TRBC2**	8.205346225	8.205346622
**TRBV21**	8.205214112	8.205214398	**TRBC3**	8.205359080	8.205359477
**TRBV22**	8.205229993	8.205230279	**TRBC4**	8.205371814	8.205372210
**TRBV23**	8.205238420	8.205238706			
**TRBV24**	8.205242976	8.205243262			
**TRBV25**	8.205247855	8.205248138			
**TRBV26**	8.205266412	8.205266698			
**TRBV27**	8.205271012	8.205271298			
**TRBV28**	8.205384686	8.205384403			

Additional data file 3C. Location of opossum TRG gene segments†.

	FR1	RSS
**TRGV1.1**	6.283942345	6.283942040
**TRGV1.2**	6.283934927	6.283934623
**TRGV1.3**	6.283921739	6.283921428
**TRGV1.4**	6.283915397	6.283915042
**TRGV1.5**	6.283907901	6.283907590
**TRGV2**	6.283928450	6.283928153
**TRGV3.1**	6.283888100	6.283887792
**TRGV3.2**	6.283879394	6.283879086
**TRGV4**	6.283884333	6.283884026
	**RSS**	**3' Splice site**
**TRGJ1**	6.283872422	6.283872327
**TRGJ2**	6.283868960	6.283868876
**TRGJ3**	6.283864852	6.283864765
**TRGJ4**	6.283864788	6.283864670
**TRGJ5**	6.283862065	6.283861969
**TRGJ6**	6.283862001	6.283861880
**TRGJ7**	6.283859745	6.283859667
	**5' Splice site**	**3' Splice site**
**TRGC**	6.283854997	6.283854682

Additional data file 3D. Location of opossum TRM gene segments†.

	FR1	RSS		RSS	3' Splice site
**TRMV1**	3.432520406	3.432520704	**TRMJ1**	3.432554017	3.432554070
**TRMV2**	3.432586139	3.432586437	**TRMJ2**	3.432622480	3.432622533
**TRMV3**	3.432640955	3.432641253	**TRMJ3**	3.432739069	3.432739125
**TRMV4**	3.432796955	3.432797253	**TRMJ4**	3.432816295	3.432816348
**TRMV5**	3.432834911	3.432835209	**TRMJ5**	3.432949925	3.432949981
**TRMV7**	3.433001637	3.433001935	**TRMJ6**	3.432979778	3.432979831
**TRMV-OR2**	2.402780971	2.402780621	**TRMJ8**	3.433124971	3.433125024
	**FR1**	**3' Splice site**			
**TRMVj1**	3.432554616	3.432554969		**5' Splice site**	**3' Splice site**
**TRMVj2**	3.432623130	3.432623483	**TRMC1**	3.432555603	3.432555866
**TRMVj3**	3.432739676	3.432740029	**TRMC2**	3.432624184	3.432624447
**TRMVj4**	3.432816976	3.432817329	**TRMC3**	3.432740589	3.432740852
**TRMVj5**	3.432950531	3.432950884	**TRMC4**	3.432818029	3.432818292
**TRMVj6**	3.432980459	3.432980812	**TRMC5**	3.432951454	3.432951717
**TRMVj7**	3.433091285	3.433091638	**TRMC6**	3.432981512	3.432981775
**TRMVj8**	3.433125652	3.433126005	**TRMC7**	3.433092190	3.433092453
	**5' RSS**	**3' RSS**	**TRMC8**	3.433126705	3.433126968
**TRMD1.1**	3.432528952	3.432528968			
**TRMD1.2**	3.432538487	3.432538498			
**TRMD2.1**	3.432588853	3.432588869			
**TRMD2.2**	3.432602249	3.432602263			
**TRMD3.1**	3.432665676	3.432665692			
**TRMD3.2**	3.432675898	3.432675914			
**TRMD3.3**	3.432701119	3.432701134			
**TRMD4.1**	3.432800121	3.432800137			
**TRMD4.2**	3.432803411	3.432803425			
**TRMD5.1**	3.432850229	3.432850245			
**TRMD5.2**	3.432863476	3.432863490			
**TRMD5.3**	3.432919820	3.432919835			
**TRMD7.1**	3.433030490	3.433030502			
**TRMD7.2**	3.433058301	3.433058316			
**TRMD7.3**	3.433068169	3.433068184			

† Locations are given according to MonDom5 assembly. Positions for V segments include from the beginning of framework 1 (FR1), as defined by IMGT, until the first nucleotide of the recombination signal sequence (RSS). 5’ RSS and/or 3’RSS positions for D and J segments refer to the first nucleotide in the heptamer of the RSS. For TRC regions the positions given correspond to exon 1.
