# Supplementary material for: Comparative genomic analysis and evolution of the T cell receptor loci in the opossum Monodelphis domestica
Source: BMC Genomics. 2008 Feb 29;9:111. doi: 10.1186/1471-2164-9-111 (PMC2275272; doi:10.1186/1471-2164-9-111)
Supplement: Additional file 4 — List of primers used to amplify opossum TCR. this table contains the sequences of forward and reverse primers used to amplify opossum TCR. [file 1471-2164-9-111-S4.pdf]

Additional data file 4. List of primers used to amplify opossum TCR†.

	PRIMER 5' - 3'		PRIMER 5' - 3'
**TRAV1**	GAGCAGCCGGCTCTGTTGATGGTC	**TRAV41**	CAAGGCCTTCAGTGGTTCAGGCAAG
**TRAV2**	AGGGGACCCATTGGAAGTGAAAT		GTCCCTGAAGATCCAGGAGGGAGA
**TRAV3**	CAGTCCATGACAGTCCAGGAGGATG		GGGCTTCAGTGGATCAGGCAGGA
**TRAV5**	GGCCTGCAATGGTATAGGCAGTATC		GGACTGCAATGGTTCAGGCAGGAT
**TRAV6**	GGGAGAAAACCAAGGAAGGGTCAG	**TRAC**	TCATCTCCAGGACTGTTGCTTCG
**TRAV7**	GGTCAAGTGAACACACGGAAGGAC	**TRDV1**	CCATCACCCTCAACGTCTTGTCCTC
**TRAV8**	AGATGGTGCTGCCCGCTACTTTGC	**TRDV2**	GCACCCTGAAAGGAGGGGACCTGA
**TRAV10**	CATCCAGGAGGGAGAGAGCATCAC	**TRDV3**	CGAAGTCTGTGGCTCTGGTTTCCA
**TRAV12**	GGGAAGATTCGCAATGCACCTCAA	**TRDV6**	GGCATTGGGCAGGGAAGTGACTCT
**TRAV17**	CAGGTATCCCAACCAGGGCCTTCA	**TRDC**	CCAGACATGCCACATTGGTCCC
**TRAV18**	CTGGGAAAGGGCCAGATTTTCAGA	**TRBV1**	GATGCAGGAATGCCCAAATCTCG
**TRAV20**	CAGCCTTCCAGTGGGGAGCTGATT	**TRBV3**	GGTATCGGCAGGATGACCAGCA
**TRAV21**	GGCAGGAACCTGGCAAACAACCAG	**TRBV4**	CCTGGCTTTCCTACAGGATAACC
**TRAV22**	GCAGATTCCAGGGCAAGGTCCAGT	**TRBV6**	CAAGGAAAGGACAGTCAGTGGCC
**TRAV23**	GGTGTCAAATGGCGCTGTGAAGCA	**TRBV8**	CCCCATGGCACTTGATCACAAGGA
**TRAV24**	ATGCACCATCTGGGATCTGCTCCA		GGTATCAACAGGATTCCAAGATGG
**TRAV25**	GATGGGAGGCACAGCCATCATCAA	**TRBV9**	GGTACCGCCAGCTTCCACAGCAG
**TRAV26**	CCTCCTCCTGAGAGGAACAGACA	**TRBV11**	CCAAGCCCATCGGTCAGAAATGA
**TRAV27**	GCATCCTGGGAAAGACCCTAGGAA	**TRBV12**	CCAGATCCCCAAACAGCTTATCG
**TRAV28**	CGGGCAAACGGGGCTCTGAAGATA	**TRBV13**	CCCCATTTCTGGACACAATGCAC
**TRAV29**	TCCAGCATCCCAACGAAGGTCTGA		GCCCCATTTCTGGACATGTTGCTC
	CCTTCCTGAGGGAGCACCATTCAC		TGGTACCGACAGGTCCCAGGGAAG
**TRAV30**	CTGCCTGGTTTGACGAGAAAAAGC	**TRBV16**	ACCAGCTTCCCCAACAGAGCTTGG
**TRAV31**	GCAGTCTCCTGGTGGGGAGCTGAT	**TRBV17**	GGTGCTCAGCTGTGATCCCATCAA
**TRAV32**	CATTGCTTCCGGGGTGAAGCAGAA	**TRBV19**	TGGGGCTGCGGCTGATTCATTATT
**TRAV33**	CAGGAGGTGGGGAAAAGCCTGAAG	**TRBV20**	GGGACAGTCCGTGACGCTGAAATG
**TRAV34**	CAGCATCCTGAGCCTGCCTCCAGT	**TRBV21**	GTCTTGACCCTCCCCATCCACACC
**TRAV35**	CAAAGGAGCGGCAAAGTTCCCTGT	**TRBV22**	TGGGATGTGAACAGACTTTGGGACA
**TRAV36**	CCCTCAATCCCTGAGCTTGCATGA	**TRBV23**	CAGGGCCAGGGGCTTCAACTCATT
**TRAV37**	GGCCTTGAGAGTCCAGGAGGGAGA	**TRBV24**	TGCTGAGGTGTGACCCAATCAAGC
	TCCCAAAGCTTGCAGTGGTTCAGG	**TRBV25**	CGACAGGACCCAGGGTTAGGGCTAC
**TRAV38**	TCCTGGGAAAGGGCTCATCCTCCT	**TRBV26**	TCGTGCCTCTAGGACAAAGCAGCA
	GGATCCTGGGAAAGGGCTCATCTC	**TRBV27**	CTGCAGTGTGAGCCAATGCCCACT
	CCCACCCAGAGTGTCCAGGAAGGA	**TRBV28**	GCAGGAAGCCAGCTTTTCCTGGAG
**TRAV39**	CAGTGCTTCCCAAGGGCTTCAATGG	**TRBC**	CTGACCCCAATTTTGGTCTCCTG
**TRAV40**	GGCCTGCAATGGTTGAGGCAGGAT	**TRGV1**	CACCTGTCCTGTGCCCACATG
	CAAGATCCTGGGAAAGGACCCACC	**TRGV2**	GTCGCCCTGGAGCAGAGGCCCATC
	CATCCCAAACTTCTCAGGGCCTCCA	**TRGV3**	CAGGCAGCCTTGAAGCAGCCTC
		**TRGV4**	CCCATGCTGGCCCAGCTCATGCC
		**TRGC**	GGAAAAAGATGGGCTTTGGTGGC

†Forward primers were designed per each TRV subgroup; however in some cases several primers were necessary to amplify all the TRV members of a subgroup.
